# Computational analyses of mechanism of action (MoA): data, methods and integration†

**DOI:** 10.1039/d1cb00069a

**Published:** 2021-12-22

**Authors:** Maria-Anna Trapotsi, Layla Hosseini-Gerami, Andreas Bender

**Affiliations:** Centre for Molecular Informatics, Yusuf Hamied Department of Chemistry, University of Cambridge UK ab454@cam.ac.uk

## Abstract

The elucidation of a compound's Mechanism of Action (MoA) is a challenging task in the drug discovery process, but it is important in order to rationalise phenotypic findings and to anticipate potential side-effects. Bioinformatic approaches, advances in machine learning techniques and the increasing deposition of high-throughput data in public databases have significantly contributed to recent advances in the field, but it is not straightforward to decide which data and methods are most suitable to use in a given case. In this review, we focus on these methods and data and their applications in generating MoA hypotheses for subsequent experimental validation. We discuss compound-specific data such as -omics, cell morphology and bioactivity data, as well as commonly used supplementary prior knowledge such as network and pathway data, and provide information on databases where this data can be accessed. In terms of methodologies, we discuss both well-established methods (connectivity mapping, pathway enrichment) as well as more developing methods (neural networks and multi-omics integration). Finally, we review case studies where the MoA of a compound was successfully suggested from computational analysis by incorporating multiple data modalities and/or methodologies. Our aim for this review is to provide researchers with insights into the benefits and drawbacks of both the data and methods in terms of level of understanding, biases and interpretation – and to highlight future avenues of investigation which we foresee will improve the field of MoA elucidation, including greater public access to -omics data and methodologies which are capable of data integration.

## Abstract 2

This review summarises different data, data resources and methods for computational mechanism of action (MoA) analysis, and highlights some case studies where integration of data types and methods enabled MoA elucidation on the systems-level.

## Graphical abstract

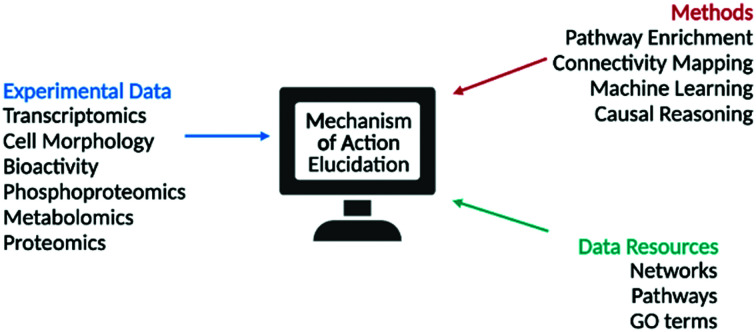

## Introduction

A principal challenge in the drug discovery process is the development of therapeutic small-molecule compounds and the understanding of their ‘Mechanism of Action’, which is the term used to describe the biological interaction through which a molecule produces its pharmacological effect.^1^ The terms ‘Mode of Action’ and ‘Mechanism of Action’ are often used interchangeably but refer to different concepts. Mode of action usually refers to the functional or anatomical changes at a cellular level induced by exposure to a substance, whereas Mechanism of Action includes specific targets or pathways modulated by the compound.^2^ Understanding the biological mechanism of a compound is important for many reasons, including the identification of toxicity or potential side-effects, or for rationalisation of a phenotypic effect to provide more confidence in a lead compound prior to clinical trial.^3^

### The importance of mechanism of action in drug discovery

Despite the many benefits of understanding a compound's Mechanism of Action, the knowledge of a drug's Mechanism of Action is not a requirement to get Food and Drug Administration (FDA) approval if the drug shows safety and some efficacy^4^ (though phase 2 testing may be shortened or skipped if the MoA is well understood^5^). For example, the compound Metformin – used in the treatment of type 2 diabetes – entered clinical trials in the 1980s,^6^ but the drug's function is still unclear, other than some proposals such as AMP-activated protein kinase (AMPK) inhibition.^7^ One example of a drug entering clinical trial with unknown MoA, which lead to unwanted consequences is the failure of Dimebon, a drug initially developed as an antihistamine for allergy treatment and later in the 1990s entered clinical trials as a potential treatment for Alzheimer's disease due to a hypothesised stabilisation of mitochondria.^8^ However, Dimebon failed to affect cognition in a large follow-up phase 3 study, and this was attributed to the lack of characterisation of its MoA. Further independent studies which have followed this phase 3 failure have identified inhibition of histamine H_1_ and serotonin 5-HT_6_ receptors as the main biological mechanisms of Dimebon.^9^ The true MoA explains the positive effects on cognition seen in the smaller-scale trials, but ultimately Dimebon did not stabilise mitochondria as first hypothesised. If this proposed mechanism was investigated more thoroughly in preclinical studies, then the failure of Dimebon could have been prevented, as they would have discovered that the observed cognitive efficacy is attributed to the engagement of histamine and serotonin receptors and not due to effective Alzheimer's disease treatment.

The concept of defining a compound's MoA is very complex if we also take into account that compounds do not only directly act on protein targets, such as in the case of alkylating agents, membrane disruptors, compounds that change the pH on an environment (or other physiochemical properties), impact transport or distribution, *etc.* They work by adding an alkyl group to the guanine base of the DNA molecule. In addition, the concept of MoA understanding is even more complicated if we take into account the emerging new data modalities such as Proteolysis Targeting Chimeras (PROTACs), which exert their effect by degrading the targeted protein rather than occupying the protein's binding site. These bifunctional molecules differ from the classic ‘small molecules’, which usually act by occupying the binding site of a target.^10^ In contrast, PROTACs bind to the protein of interest with one end while the other end binds to an E3 ligase and thus work through the active recruitment of an E3 ligase in order to tag proteins for disposal.

### A systems view of mechanism of action

**Fig. 1:**
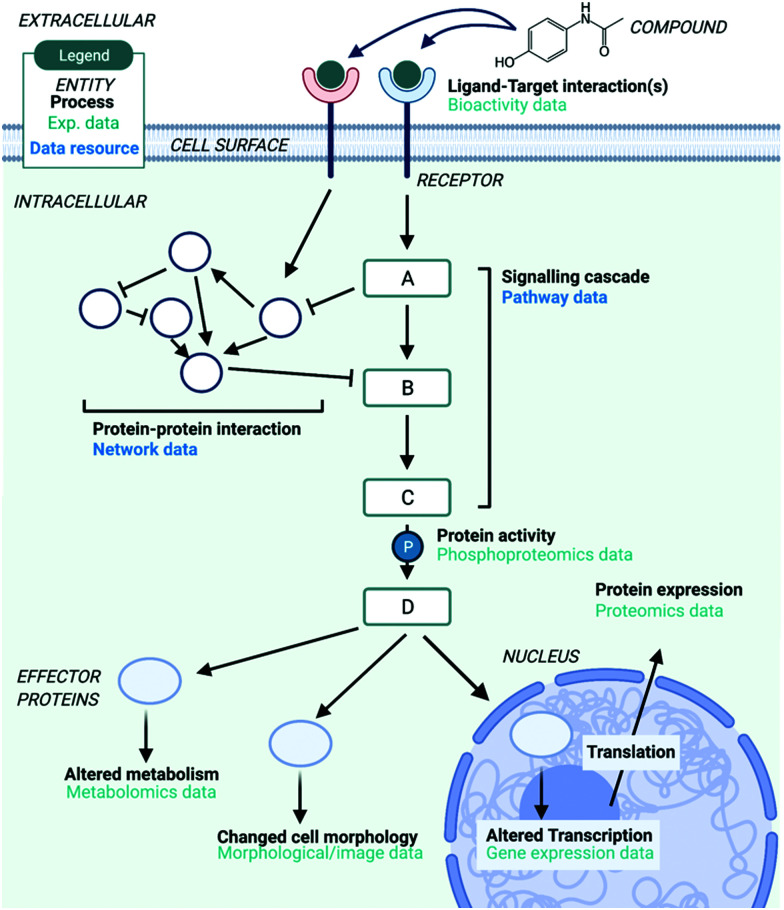
Overview of the different types of data/information used in MoA studies and the various levels that MoA can be defined on, as reviewed in this paper. This includes experimental data, such as transcriptomics data, and data resources which are used to provide biological context to experimental data, such as pathway and network data. Created with BioRender.

The story of Dimebon underlines the importance of MoA studies in the development of new drugs – however, the concept of MoA can be defined on multiple levels of biology which makes this challenging (illustrated in Fig. 1). Although a compound's MoA could be defined as the direct target(s) it interacts with, this is a relatively ‘shallow’ level of detail – after target engagement a number of signalling proteins can be differentially regulated through cellular signal transduction, leading to changes in transcription, translation, metabolism and cell morphology.^11^ Following the modulation of protein(s) by direct pharmacological action, cellular signalling proteins propagate signals *via* protein phosphorylation,^12^ catalysed by enzymes called kinases. These signalling cascades form pathways, which lead to a cellular response through the modified activity of so-called ‘effector’ proteins.^13^ Signalling pathways can also interact with each other *via* ‘cross-talk’, forming networks and a coordinated cellular response.^14^ Thus, a compound's Mechanism of Action can be defined on the systems-level in terms of the pathways that are modulated (signalling proteins), network perturbation, or by changes brought about to the cellular response (effector proteins) – and to further complicate things, the precise response will vary in different cells and tissues due to different patterns of protein expression.^15^

It is therefore advantageous to broaden further from target identification to gain a systems-level view of compound mechanism in terms of the signalling proteins and effectors it modulates, as a consequence of target engagement. For example, consider the anti-breast cancer drug Trastuzumab which binds to the epidermal growth factor receptor HER2, expressed at very high levels in some breast cancers. The knowledge that Trastuzumab modulates the PI3K/AKT pathway leading to reduced cell growth and proliferation *via* binding to HER2 gives further mechanistic insight into the anti-cancer actions of the drug.^16^ Furthermore, Trastuzumab-resistant HER2-positive breast cancers can no longer be treated by modulating HER2 due to a mutation in the binding site. Instead, the same pathways can be modulated *via* a different entry point (*e.g.*, another upstream target), paving the way for more successful patient-stratified breast cancer treatments. The MoA of Trastuzumab independent of HER2 could also be related to antibody dependent cell cytotoxicity (such as Dinutuximab). This illustrates that going beyond understanding on the target-level to the systems-level can help to better rationalise the observed phenotypes induced by a compound, and allow for personalised treatment strategies.

### Bioinformatics approaches to understanding mechanism of action

These different levels of biology which define a compound's MoA on the ‘systems-level’ can be captured and measured with different types of data, such as transcriptomics, cell morphology and metabolomics data (Fig. 1), all of which provide a different aspect of the bioactivity of a compound. Additional information which catalogues known human pathways and networks can also be useful as supplementary prior knowledge to contextualise different types of data – for example, by relating differentially expressed genes to the pathways they participate in. To better understand the MoA of compounds the use of a combination of different types of biological data can be very enlightening, in particular since the insight gained from different types of information can differ greatly. For example, two structurally similar compounds, the antidiabetic drugs rosiglitazone and troglitazone, exhibit a very different side effect profile due to their different MoAs.^17^ Both compounds belong to the thiazolidinediones class and treat insulin resistance in type 2 diabetes mellitus. Troglitazone was withdrawn from the market because of hepatotoxicity and rosiglitazone was developed as an alternative, which has been linked with cardiovascular diseases. The exact mechanistic reasons behind those adverse side effects are not fully understood. A recent study docked the two compounds into predicted binding sites of more than 67 000 protein structures.^18^ Targets of troglitazone such as 3-oxo-5-beta-steroid 4-dehydrogenase, neutrophil collagenase and others could explain why troglitazone causes hepatotoxicity. Results for rosiglitazone discerned its interaction with members of the matrix metalloproteinase family, which could lead to cancer and neurodegenerative disorders. The concerning cardiovascular side effects of rosiglitazone could also potentially be explained. In two recent studies transcriptomic data and data that capture the changes in cell morphology upon compound perturbation have been shown to be complementary to chemical information in target prediction;^19,20^ gene expression data outperformed chemical-based information in target prediction models for 25% of the targets and cellular morphology information outperformed chemical based target prediction models for 40% of the targets. In addition to these findings, the evaluation and generation of multi-omics data highlights that we can approach compounds’ MoA from a more holistic molecular perspective.

To generate hypotheses for compound MoA for further experimental validation, these data can be harnessed with various computational algorithms. Approaches such as machine learning, pathway enrichment, connectivity mapping and causal reasoning can harness -omics data as well as prior knowledge such as protein–protein interaction data to infer both compound targets and signalling proteins. Additionally, each computational method has different considerations which will be discussed in this review such as the type of input data required, computational time and complexity, which must be considered when choosing which method is most suitable for the particular compound(s) in question and the level of understanding which is desired.

In this review, we shall first outline the data which captures different levels of biology relating to compound MoA (what is captured, and what are the advantages and disadvantages of this data), including examples of public resources which allow access (or improved interpretation) to this data as described in Section 2. In the following section, we review the most prevalent methods that are employed to leverage this data in the understanding of MoA, some considerations (*e.g.*, limitations and biases) and some examples of the methods being implemented in open-source software packages. In the final section we outline some case studies where researchers have combined different data sources and methods to more comprehensively understand the MoA of compounds on different levels of biology, supporting our view that it is necessary to interrogate MoA on multiple levels to get a more comprehensive understanding of this very complex concept.

## Data and databases for mechanism of action elucidation

It can be seen from Table 1 that each type of data captures a different aspect of a compound's MoA – for example, transcriptomics data describes differential mRNA expression following compound perturbation, while bioactivity data describes the protein receptor(s) that the compound directly binds to, and network data provides prior knowledge in the form of known cellular protein–protein interactions. This enables complementary types of data to be integrated – such as phosphoproteomics data, which describes differential protein signalling induced by compound perturbation, and pathway data which catalogues signalling proteins into biologically interpretable signalling cascades or pathways. The different advantages and limitations of these data types, as well as databases which contain this data, will now be discussed to facilitate MoA elucidation on the systems-level.

**Table 1:** Experimental data types commonly used in MoA analysis, the level of biology represented, and some advantages and disadvantages of the data, which are usually generated with high-throughput unbiased techniques

Data type	MoA biology represented	Advantages	Disadvantages	Experimental techniques
Bioactivity	Compound-target binding and functional effects (*e.g.* activation, inhibition)	Relatively easy and cheap to measure (high-throughput screening or HTS)^21^	Target binding *in vitro* is not necessarily indicative of target engagement *in vivo* due to *e.g.* ADME/PK effects^22^	There is a broad spectrum of assays to test bioactivity of compounds to targets.
			Does not inform about specific changes in cell signalling following target binding	*E.g.*, direct biochemical methods, genetic interaction methods^23^
			Not all target–ligand interactions are efficacy (MoA) related (*i.e.* could be side-effect related)	
Transcriptomics	Changes in gene expression arising from modulated signalling (and transcription factor activity)	High-throughput techniques developed for large data acquisition^24^	High level of noise in data arising from fluctuations in biological activity^25^	Micro-array and RNA-sequencing
		Provides a ‘snapshot’ of cellular changes in signalling following compound administration	Assumes gene expression is static, rather than a dynamic process^26^	
		An array of standard analysis methods have been developed	Does not necessarily translate to protein expression due to *e.g.* post-translational effects^27^	
Cell image	Changes in cellular morphology (*e.g.* size and shape of organelles) arising from modulated signalling (and changes in cytoskeletal protein activity)	High-throughput imaging techniques developed for large data acquisition^28^	May not produce a meaningful signal if the compound is not able to alter cell morphology.^31^	High throughput imaging assays
		Feature extraction software and methods are evolving^29,30^	Features are often highly correlated and biologically ambiguous^32^	*E.g.*, cell painting
		Young field	Requires orthogonal data to be able to relate changes to modulated genes/proteins^28^	
		Little case studies for MoA analysis	Phenotypic effects may be subtle and hence the biological signal can be overwhelmed by sources of technical variation^28^	
Proteomics	Changes in protein abundance arising from modulated signalling induced by a compound (transcription, translation, protein degradation)	Extends upon transcriptomics data by capturing changes in post-translational regulation	Data generation is costly and cumbersome^33^	LC-MS/MS
			High biological variability/low reproducibility as well as significant technical variability^33^	
			‘Missing value problem’^34^	
Metabolomics	Changes in metabolite abundance arising from modulated signalling induced by a compound (and metabolic enzymes)	Contains downstream products of transcriptomic and proteomic processes^35^	Data generation is costly and cumbersome, requiring multiple technical methods to capture the entire metabolome^35^	NMR, LC-MS
		Can also identify potential toxicity^36^	High biological variability/low reproducibility as well as technical variability due to *e.g.* long sample runs^37^	
			Lack of comprehensive metabolite annotation and ability to relate to other biochemical components (*e.g.* enzymes)^38^	
Phosphoproteomics	Changes in protein phosphorylation (protein signalling) induced by a compound	Captures the signalling proteins modulated, thus the specific biological pathways relevant to MoA	Phosphorylation site annotation is not trivial and functional relevance is often unclear^40^	MS
		Links ‘higher-level’ bioactivity data and ‘lower-level’ *e.g.* transcriptomics data, enabling a ‘systems-view’	Time-consuming assays limiting data availability^41^	
		High-throughput assays in development^39^	High biological variability/low reproducibility, as well as technical variability arising from MS instruments^39^	

### Bioactivity data

Compound-target activity, or ‘bioactivity’ data distils target binding into a numerical value, usually in terms of a concentration where target activity is seen (or % of some functional effect such as target inhibition) (Table 1). This data can be highly valuable in MoA studies as it can be used to predict targets for orphan compounds,^42^ or to inform about drug repurposing opportunities.^43^ High-throughput screening (HTS) technologies have been developed which enable rapid and cheap screening of thousands of molecules against panels of compound targets,^21^ thus large-scale databases of bioactivity measurements are available.^44,45^ However, *in vitro* target binding is not necessarily indicative of target engagement *in vivo*, due to how compounds are absorbed, metabolised, metabolised and excreted (ADME) in a biological system, governed by the compound's pharmacokinetic (PK) properties.^22^ This is indeed relevant to any type of biological data measured *in vitro*, but attempts have been made to consider this in bioactivity data by utilising experimental properties such as maximal blood concentration (CMax) and plasma protein binding (PPB).^46^ Furthermore, this can be considered a relatively ‘shallow’ level of data, due to the fact that it does not inform about any changes in the many cellular signalling pathways which can be modulated following target binding, and hence the relationship between binding and a functional effect of interest needs to be determined. Additionally, target binding may not necessarily be indicative of MoA, as the so-called ‘promiscuity’ of some compounds means that they may bind to many ‘off-targets’.^47^

Bioactivity data can be accessed publicly in databases such as ChEMBL, PubChem, ExCAPE and BindingDB (Table S1, ESI†). ChEMBL contains more positive/active data points because data are derived from literature, compared to PubChem, where there is a plethora of negative bioactivity data from HTS. The ExCAPE (Exascale Compound Activity Prediction Engine) database is an integrated version of ChEMBL (version 20) data and PubChem data (extracted in January 2016).^48^ It is important to mention that data in these public databases are extracted from different publications and data were prepared in various laboratories and with different assays. Hence, there is expected that there is a degree of experimental uncertainty in the data (0.47 log units for mixed p*K*_i_ data in ChEMBL v14)^49^ and this experimental uncertainty sets the upper limit of performance that can be achieved from *in silico* target prediction models. Beyond experimental error in data, another parameter that should be taken into account is the chemical space coverage of the chemical structures in the bioactivity databases. Despite the fact that millions of chemical data have been deposited in such databases (*e.g.* more than 15 million bioactivity data points for ∼2 million compounds, including compound interaction data against ∼8000 protein targets in ChEMBL) and the exponential increase of such data because of the application of parallel and combinatorial synthesis approaches, the available data corresponds only to a small part of chemical space of all possible molecules.^50^ This is a parameter that should be considered when extracting and using data for projects that focus on ‘poorly explored’ areas of chemical space. For a review on bioactivity data in mechanism of action studies, see ref. 23.

### Transcriptomics data

Transcriptomics data informs about changes in transcription factor activity in terms of differential mRNA abundance, providing a ‘snapshot’ of cellular signalling, and is thus very valuable for compound mechanism of action analysis (Table 1). High-throughput techniques such as the L1000 assay,^24^ DRUG-Seq^51^ and TempO-Seq^52^ have been developed for large amounts of data acquisition, and standard analysis pipelines for data processing have been developed.^53^ Recent advances in single-cell technology have enabled the capture of cell type-specific changes in gene expression, as opposed to the traditional bulk tissue-level measurements.^54^ Due to the “stochastic or inherently random nature” of the biochemical reactions of gene expression, there is some variability in gene expression data, leading to a degree of noise, which can be dealt with by performing experiments in replicates and correcting for batch effects (if needed).^25,55^ This limitation is applicable to all types of ‘high-dimensional’ biological data due to both the nature of biology as well as technical variation. Furthermore, transcriptional changes are a dynamic process, but gene expression data only captures a static snapshot at a particular point in time, thus measurements are often taken at different time points to capture temporal changes in gene expression induced by a compound.^26^ Additionally, the choice of *in vitro* cell line or cellular model, as well as treatment concentration and dose, are important parameters and should ideally be chosen for concordance to *in vivo* treatment in question – for example by choosing a biologically relevant cell line, and concentrations/time points relevant to predicted or measured ADME properties. Transcriptomic changes are often assumed to be equivalent with changes in protein expression, but the correlation between the two is often very low – no more than 0.5 on average,^27^ based on baseline cellular measurements. However, correlations between differentially expressed genes (DEGs) and their protein products following compound administration are a more important thing to consider for MoA studies. For example, a study in an ovarian cancer xenograph model found a significantly higher correlation between mRNA and protein for DEGs *vs.* non-DEGs, indicating the usefulness of this data type for biological discovery.^56^ Nevertheless, other processes such as post-translational effects which regulate protein abundance are not captured with gene expression data. With regards to data from the L1000 assay, the selected landmark genes were chosen to for imputation (rather than for biological discovery), hence the measured genes may not necessarily be optimised for MoA analysis. The imputation itself is performed using linear regression, which does not capture non-linear relationships between genes, so improved techniques for inference of non-landmark genes based on deep learning have been suggested as an alternative.^57^

The main freely available sources of gene expression data are CMap,^58^ LINCS,^24^ GEO,^59^ ArrayExpress,^60^ DrugMatrix^61^ and Open TG GATEs^62^ (Table S2, ESI†). The L1000 assay measures the expression of only 978 ‘landmark genes’, and inferring the rest of the transcriptome based on a correlation analysis of the underlying gene expression structure. The LINCS dataset is a follow-up of the CMap dataset (which is no longer updated), which has been measured with traditional microarrays, and which aimed to build a comprehensive and freely available database of gene expression signatures in multiple cell lines for mechanism of action studies (‘connectivity mapping’). The LINCS database primarily contains chemical perturbants, as well as genetic (*e.g.* shRNA knockdown). Despite the quantity of data in the LINCS database, concerns have been raised about the quality of the data, in particular due to the low reproducibility of data derived from the L1000 platform *vs.* matched-condition microarray data, and even within-platform replicates. This was found to affect downstream analysis in drug repositioning.^63^ The Gene Expression Omnibus, or GEO, also contains user-submitted, publicly available gene expression data for a variety of perturbants including disease, gene and compound, measured with differing platforms (RNA-Seq, microarray) and in different species. GEO contains the most samples overall, but the LINCS database contains data measured and processed with the same protocol, which can be beneficial when harnessing high-throughput data to avoid confounding factors arising from technological differences (“batch effects”). ArrayExpress contains curated, well-annotated and reproducible gene expression data (both RNA-Seq and microarray), again with perturbants covering both compounds and diseases. Two ‘toxicogenomics’ databases; that is, databases containing transcriptional data for toxicology research, which can be useful for mechanism of action studies are DrugMatrix and Open TG GATEs. These databases contain data about a small number (600 and 170, respectively) of compounds including both pharmaceuticals and industrial/environmental chemicals both *in vivo* and *in vitro* and across multiple doses, though these are primarily measured in rats – thus human concordance must be considered if relevant. For a review of transcriptomics data in MoA studies, see ref. 64.

### Cell image data

**Fig. 2:**
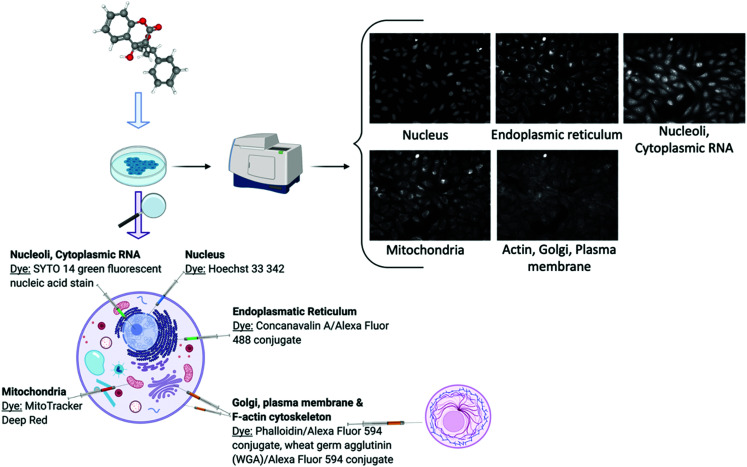
Schematic description of the cell painting assay demonstrated with the Warfarin compound. Created with BioRender using cell images from the Image Data Resource (IDR0036).

Cell image or cell morphology data captures the morphological changes which occur when a chemical compound is applied on cell cultures, due to *e.g.* changes in cytoskeletal protein activity or apoptosis^65^ (Table 1). Such data can depict any cell morphological characteristics upon compound perturbation and hence readouts have a general nature, being particularly popular in toxicology research.^66^ Recently, new assays have been developed for large data acquisition, such as the Cell Painting assay^28^ (Fig. 2) which measures morphological changes in organelles or cellular sub-compartments which have been fluorescently stained with different dyes. Computer vision has been successfully employed to cell segmentation and feature extraction and a prominent example of this is CellProfiler.^67^ CellProfiler is an open-source software that measures and analyses cell images. In addition to CellProfiler, other segmentation programs are the CellCognition^68^ and PhenoRipper^69^ and outputs from these platforms typically contain hundreds to thousands of different features for each object and image. Although these methods are mostly applicable to 2D images, new tools are being developed to extract features from 3D images as well.^70^ Furthermore, automated feature extraction methods have been under much development recently, such as Convolutional Neural Networks (CNNs)^30^ and generally deep learning can deal with diverse problems in the processing and image-based profiling. Deep learning is able to process raw microscopy images and produces representations that could be better suited for downstream analysis and interpretation because cells or cellular subcompartments or substructures can be identified more accurately.^71,72^ As a result, improved image-based descriptors can be derived and thus eventually replace the standard currently used software such as CellProfiler.^30,73^

One of the main disadvantages of image-based data is that not all compounds are able to change cellular morphology.^31^ Therefore, it is important to select compounds for downstream analysis that are considered to be ‘active’ on the image assay – *i.e.*, compound's image-based profiles are significantly different from the control wells. This process involves arbitrary cut-offs to define how different a compound is to the control wells such as Euclidean distance.^74^ In addition, when curating image-based data it is important to evaluate potential intra or inter plate effects as well as the reproducibility between replicate measurements.^75^ This is particularly relevant for morphological end-points because phenotypic effects may be subtle, hence the effect of technical variation may overwhelm any biological signal in the data.^28^ Furthermore, cell morphological features can often reflect technical properties of the image rather than biological characteristics of the cell, and there is high redundancy among morphological features.^32^ Finally, when using such data for Mechanism of Action understanding, it is not trivial to link particular morphology-based markers or features to modulated signalling proteins or targets. To facilitate biological interpretation of cell image data it is recommended that orthogonal and complementary assays (*e.g.* transcriptomics) be carried out in tandem.^28^

A variety of image-based datasets have been developed and deposited in public repositories (Table S3, ESI†) such as the Broad Bioimage Benchmark Collection (BBBC) developed by the Broad Institute^76^ and other databases such as the ‘Cell Image Library’ and the Image Data Resource (IDR).^77^ A large dataset of 30 616 compounds was released in the GigaScience database by Bray *et al.*,^78^ including a variety of perturbations (drugs, natural products, small probe molecules, diversity-oriented synthesis compounds) and numerical image-based features/descriptors. There is a joint effort from Imaging Platform at the Broad Institute of MIT and Harvard with 12 industry and non-profit partners with the aim to release a large reference collection of image data with 1 billion cells responding to over 140 000 small molecules and genetic perturbations, which will greatly benefit researchers seeking access to this data type.^79^ Moreover, Recursion Pharmaceuticals is focusing on combining high-content phenotypic screening with machine learning for emerging opportunities in target discovery, and hit identification, releasing their datasets in the public domain.^80^ For reviews on the use of cell image data for MoA analysis, see ref. 30 and 75.

### Proteomics data

Proteomics data measures changes in protein abundance (due to modulation in translation or degradation) arising from compound-induced protein signalling^81^ (Table 1). Proteomics data is complementary to transcriptomic data as it informs about cellular processes following transcription, such as translation and post-translational modifications. By studying interrelationships of protein expressions and modifications following a drug treatment, important insights of a compound's Mechanism of Action, toxicity and side effect profile can be identified.^82^ Therefore, the knowledge about which proteins are differentially expressed due to a compound treatment can inform researchers about the proteins which are key to its mechanistic action. Due to technological limitations (LC-MS/MS measurements can take several days or even weeks to run), data generation is costly and cumbersome, and leads to biological variability between replicate measurements (due to *e.g.* decay in performance of columns over the course of a long experiment).^33^ Another limitation of proteomics data is that not all proteins are quantified in all experiments (missing value problem), though this can be addressed by using data derived from multiple assays to obtain a larger coverage of the proteome^83^ or through imputation.^34^

The PRoteomics IDEntifications (PRIDE) database is the largest data repository of MS-based proteomics data and serves as one of the most widely used platforms to deposit public proteomics^84^ (Table S4, ESI†). Another dataset, which was created with the aim to better understand the MoA of 56 anticancer compounds, is ProTargetMiner.^81^ It includes chemical proteomics data generated to study the relationship between the anticancer drug molecules and the dying cell phenotypes induced by these molecules. Another key source of proteomics data is ProteomicsDB, which published the first draft of the human proteome and allows for the exploration and retrieval of “protein abundance values across different tissues, cell lines, and body fluids *via* interactive expression heat maps and body maps”.^85^ Reviews on the applications of proteomics data and MoA analysis can be found at ref. 86 and 87.

### Metabolomics data

Metabolomics data captures the presence of metabolites (small molecules <1500 Da), and thus primarily captures perturbations to metabolic enzyme activity induced by a compound as a “functional readout of the physiological state”^88^ (Table 1). Changes on the mRNA level (transcription), lead to changes in translation and protein expression (proteomics), including the expression of enzymes involved in metabolism, thus metabolomics is a complementary source of data which can be integrated with other data types to gain a deeper understanding of MoA on a systems-level.^89,90^ Furthermore, as some metabolites are considered to be toxic, metabolomic data can inform about potential off-target effects of a compound to infer its potential safety, or to understand the metabolic pathways perturbed by the compound.^36^ Similarly to proteomics data, the main drawback of metabolomics data is that experimental methods are subject to technological limitations – for example multiple methods are required to capture the entire metabolome,^35^ and difficulties in metabolite deconvolution due to similar fragmentation patterns in mass spectrometry measurements^91^ as well as a lack of comprehensive metabolite annotation,^38^ this is known as the ‘greatest bottleneck’ of metabolomics data interpretation.^92^ Again, the metabolome is highly variable and thus must be accounted for by performing replicate experiments – and untargeted approaches performed in different labs have shown wide variation due to experimental variation arising from long sample runs.^37,93^ Tools such as PhenoMeNal and MetaboAnalyst, which contain representative datasets and standard data formats and pipelines, allows for improved reproducibility for metabolomics data (on the processing level) which is beneficial for data sharing.^94,95^

MetaboLights^96^ (EBI database) is a supplementary database for metabolomics experiments (Table S4, ESI†). It covers metabolite structures and their reference spectra as well as their biological roles, which is useful for annotating metabolomics data. It also contains a small repository of metabolomics data (715 studies, or which 212 are in *Homo sapiens*), but this spans a range of model organisms and is not focused on compound mechanism of action, thus there is not much compound-perturbed metabolomics data in this resource. EcoPrestMet^97^ is a public resource which can be used for mechanism of action studies, as it profiles the metabolome of 1279 compounds – however, these measurements are undertaken in *E. coli*. This resource was created in response to the database by Fuhrer *et al.* which measures the metabolome following >3800 gene deletions,^98^ also in *E. coli*. These two resources could thus be useful to understand the mechanism of action of compounds, and in particular their potential toxicity, in the *E. coli* model system. A review on the use of metabolomics data for MoA discovery can be seen at.^99^

### Phosphoproteomics data

Phosphoproteomics data captures changes in the phosphoproteome; the phosphorylation states of signalling proteins (Table 1). Cellular signalling is mediated by protein phosphorylation on serine, threonine and tyrosine residues,^12^ thus by understanding the changes in phosphorylation states of signalling proteins following compound treatment we can infer potential pathways modulated by the compound, beyond information that is visible on the transcriptional and translational level alone. Phosphoproteomics data is particularly useful in -omics studies as it allows us to build up a “systems-level” view of compound mechanism of action by filling in the gaps downstream of target binding and upstream of changes to effector proteins (*e.g.* transcription factors, which is reflected in transcriptomics data). One limitation of phosphoproteomics data is that the annotation of phosphorylation sites is not trivial due to for example the presence of multiple serine, threonine and tyrosine residues in one peptide.^40^ To address this limitation, services such as PhosphoSitePlus®^100^ have been developed which map phosphorylation sites to proteins, and provide biological context through disease and pathway annotations. Also protein enrichment is required before quantification (for a review on common techniques, see ref. 101), which introduces variability from differences in experimental design. Furthermore, phosphoproteomic profiling of compounds is time consuming and expensive^41^ – though this has been addressed with the P100 assay, which measures only 100 phosphorylated peptides from cellular proteins and thus serves as a reduced representation of the phosphoproteome.^39,41^ Similarly to its transcriptomics analogue L1000, the inference of the rest of the phosphoproteome from the measured 100 peptides remains a challenge; however in this case the reduce phosphoproteome was derived from drug-treated data (and hence more relevant for MoA discovery compared to the L1000 landmark genes). Furthermore, much like changes to transcription, metabolism and translation, phosphorylation changes are highly variable, and there is added technical variability arising from MS instruments, hence replicate experiments are necessary to ensure the reliability of the data.^39^ A review on the use of phosphoproteomics data for MoA analysis can be found at ref. 102.

The P100 dataset is a reference phosphoproteomic signature resource in response to compounds (Table S4, ESI†). In more detail, 90 small molecules with a spanning MoA with focused subsets of kinase inhibitors and epigenetically active compounds were profiled.^41^ It is the first public data resource of proteomic responses that extends the ‘connectivity map’ concept to phosphoproteomics. The samples were profiled with a reduced-representation phosphoproteomic assay (called P100).

The above readouts can be measured from CRISPR experiments, which is a complementary approach for MoA understanding. This is usually performed by parallel integration of gene loss-of-function screens with drug response in order to investigate drug-mechanism of action. CRISPR–Cas9 based functional genetic screens have been proven to be successful methods for identifying drug targets.^103,104^ CRISPR based approaches enable one to readily repress, induce, or delete a given gene and determine the resulting effect on drug sensitivity.^105^ For example, Gonçalves *et al.* illustrated how integrating cell line drug sensitivity with CRIPSR loss-of-function can elucidate MoA.^106^ They revealed a positive association between mitochondrial E3 ubiquitin-protein ligase MARCH5 dependency and sensitivity to MCL1 inhibitors in breast cancer cell lines and estimated drug on- and off-target activity. CRISPR screening data have become available for various cell lines in the form of transcriptomics, cell morphology data and others. Compound profiling across panels of cell lines can be performed, and so this approach could become a routine step in drug discovery pipeline. CRISPR screens have utility during the hit-to-lead or lead optimisation stages of drug development to select compound series with optimal potency and selectivity. It could also be combined with orthogonal experimental (such as kinobead assays) or computational approaches (*e.g.* docking, target prediction). For more detailed information on how CRISPR technology is being integrated in drug discovery process, we are recommending some recent review papers.^107,108^

As well as compound-specific data described above, bioinformatics approaches can be carried out with prior knowledge of biological pathways and interaction networks, to relate inferred genes, proteins and other molecules with what is currently known about different biological processes. Such prior knowledge or supplementary data is usually derived from experiments, but some databases feature inferred or predicted protein–protein interactions to improve coverage. The two main types of supplementary data, Network and Pathway data, are summarised in Table 2.

**Table 2:** Supplementary data commonly used in MoA analysis, the level of biology represented, and some advantages and disadvantages of the data

Data type	MoA biology represented	Advantages	Disadvantages
Network	Global interactome of molecular entities (*e.g.* proteins) and the interactions between them	Can be used as prior knowledge with *e.g.* transcriptomics data to gain insights into compound MoA^109,110^	High false positive and false negative rates for interactions (*e.g.* protein–protein) and other technical limitations such as cost and lengthy experiments^116–120^
		Standardised formats have been developed for effective data integration and sharing in line with FAIR principles^111–113^	Curation bias – well-studied entities usually ‘hub’ nodes which bias downstream analyses^117^
		Interaction filtering is possible based on types of evidence, allowing for greater flexibility^114,115^	Simultaneously noisy and incomplete^121^
Pathway	Describes cascades of molecular interactions which have a defined entry point, signalling mediators, and cellular effect	Enables groups of genes/proteins to be characterised in terms of shared biological functions for ease of interpretation^122^	Static representation of a dynamic process^123^
			Interactions between pathways often not considered^124^
			Curation bias – well-studied processes more comprehensive and detailed, and overrepresented in pathway databases^125^

### Biological network data

Biological network data aims to capture the interactome of physical molecular interactions (Table 1), often used as a supplementary source of prior knowledge along with experimental data such as -omics data to gain new insights into the phenotype of interest on the systems’ level,^126^ making it a powerful source of data for computational mechanism of action studies.^23^ Network nodes are molecular entities such as proteins, genes or metabolites, and edges are interactions between them, which can either be directed or undirected, signed or unsigned, and entities of interest obtained from -omics data can therefore be ‘mapped’ onto a network and their interactions analysed in more detail.

The main types of biological interaction networks relevant for mechanism of action studies are protein–protein (capturing protein signalling), metabolic (describing cellular metabolic processes, including enzymes and metabolites) and transcription factor-gene (TF-gene) regulatory networks (detailing how transcription factors regulate gene expression). Proteins are at the centre of all three of these biological network sub-types, as they are cellular mediators of signalling which can interact with other proteins, genes and metabolites, and are hence key for understanding compound mechanism of action on multiple levels. Protein–protein interaction data is usually obtained from experiments such as yeast two-hybrid (Y2H) screening^127^ or affinity purification-mass spectrometry (AP/MS).^128^ AP/MS approaches have relatively high false positive and false negative rates,^116^ and Y2H approaches may identify interactions which do not actually occur *in vivo*.^117,118^ Notably, studies have shown that interaction derived from the two methods have a relatively low degree of overlap – for example, out of 80 000 interactions between yeast proteins, only around 2400 of these were supported by more than one methodology.^129^ Metabolic networks are constructed based on *in vitro* enzyme assays, which measure the activation or inhibition of metabolic enzymes upon interaction with metabolites,^130^ or *in vivo* time-course nuclear magnetic resonance (NMR) studies to elucidate and measure metabolite concentration over the course of a reaction.^119,131^ However, due to the costly and time-consuming nature of such experiments,^119,120^ mathematical modeling over metabolite abundance data has been used for the reconstruction of metabolic interaction networks. Transcription factor-gene regulatory networks can be constructed from a number of high- or low-throughout experiments such as protein binding microarrays (PBM, *in vitro*, high-throughput), MITOMI (*in vitro*, mid-throughput) and in particular chromatin immunoprecipitation combined with promoter DNA microarrays (ChIP-Seq, *in vitro*, low-throughput),^132^ which identify TF binding sites genome-wide, from which the regulated genes are inferred by mapping the DNA fragments to the relevant genome. The main disadvantage of ChIP-Seq methods is the high expense associated with reagent and sample costs,^133^ which in turn will limit the availability and coverage of TF-gene interactions, though as sequencing costs decline this will become less of a bottleneck for the availability of public transcriptional regulatory interaction data.

Biological networks in general have been described as both incomplete (low coverage of all potential interactions) and noisy (high number of false positive interactions).^121^ Different types of network display different limitations – for example, PPI data is incomplete, compared to the more complete TF-gene networks. The missing data issue has been addressed by ‘filling in gaps’ with other methodologies for interaction determination, such as gene-fusion and computational prediction for protein–protein, stoichiometric modelling for metabolic and RNAi/knockouts and computational prediction^134^ for TF-gene interactions. Furthermore, interactions are biased towards entities which have high abundance, or that participate in well-studied processes such as cancer, leading to the presence of ‘hub nodes’ in biological networks which may bias downstream analysis.^117^ Another notable limitation of all three network sub-types is the presence of protein complexes – these can be dealt with in multiple ways, such as mapping all edges to all proteins present in complexes, only the protein which physically interacts, or keeping the entire protein complex as a distinct node.^135^ To reduce noise in biological networks, interaction confidence scores have been developed to weight edges based on the inferred accuracy of each interaction, by taking into account the source (*e.g.* experiment or prediction^115^). Additionally, initiatives such as NDEx (Network Data Exchange)^111^ and IMEx (International Molecular Exchange)^112^ have enabled researchers to efficiently share and integrate network data in standardised formats which ensures compatibility with FAIR (findable, accessible, interoperable, reusable) principles.^113^ It is important to keep in mind the context of the research question and whether large-scale networks are suitable in terms of the cellular context – if the study is focused in, for example, liver cells then it is possible that many of the interactions in a global interaction network would not occur in a liver cell. In this case it is possible to constrain interaction networks based on measured RNA- or protein-level expression in the cell or tissue of interest using the Human Protein Atlas,^136^ or to consult tissue-specific databases such as TissueNet.^137^

Different biological network databases are constructed from a variety of sources (Table S5, ESI†), including in-house experiments, literature mining, and compilation of individual network databases. Individual discussion of each database is beyond the scope of this review, and have been compared previously.^138–140^ Separate databases exist for each network interaction type, for example STRING^114^ (protein–protein), RECON^141^ (metabolic) and DoRothEA^115^ (TF-gene), and different interactions types have also been combined in composite networks such as OmniPath^142^ and BioGRID.^143^ The optimal choice of network is also dependent on the specific question being asked, how the network will be analysed, and which types of interaction data are required. The aforementioned interaction confidence scores can be used to derive interactions of interest – for example, STRING allows for interaction filtering based on those derived from experiments, text-mining or predictions, while DoRothEA provides summary confidence scores based on the number of supporting evidence types. In general, if high-confidence interactions are required, then interactions derived from experiments or manual curation (BioPlex,^144^ HPRD^145^) are preferential, *e.g.* in comparison to those derived based on homology or other computational approaches. As well as filtering for interaction confidence, tissue-specific networks can be obtained from GIANT^146^ or TissueNet, in case a particular tissue of interest is being studied.

### Biological pathway data

Pathway data outlays cascades of molecular interactions which have a defined entry point and cellular effect (Table 2), for example the JAK-STAT pathway which begins with the modulation of JAK and ends with apoptosis and cell cycle progression.^147^ Pathway data is often used to supplement compound-specific data (*e.g.* transcriptomics or (phospho)proteomics), as a source of prior knowledge to enable biological interpretation of the data.^122^ Pathway data is useful for MoA studies as it links genes/proteins to observed phenotypes and is thus easily interpretable by bench biologists – if, for example, a compound induces differential expression of a set of genes known to participate in a certain pathway, then it can be inferred that this pathway is involved in the compound's mechanism of action.

Pathways have in common with networks in that they describe cellular molecular interactions, but they are much more simplified in that they aim to capture a particular cellular process rather than a global interactome network. One pathway may contain – depending on the particular pathway annotation used – interactions of multiple different types between entities, such as phosphorylation, transcriptional regulation and degradation. This raises questions about how representative such pathways truly are of the processes they aim to recapitulate, as active entities in a pathway are highly dependent on cell type and context, and they additionally act in a dynamic fashion, while pathways are usually represented as static, standalone processes.^123^ Nevertheless, for convenience and ease of interpretation pathways are represented as a ‘snapshot’ at a given time as governed by the information source the data is mined from, so this must be kept in mind when generating hypotheses using pathway annotations. Additionally, no information on their interactions is taken into account – pathways do not function independently in biological systems^124^ therefore these interactions are being catalogued in the public domain to address this shortcoming.^148^ Finally, curation bias is also present in pathway data – well-studied processes have more complete or detailed annotations and are also more over-represented in databases, hence again leading to bias in downstream data analysis.^125^

Different sources of pathway data (Table S6, ESI†) have been previously reviewed Chowdhury *et al.*,^123^ where each source was comprehensively analysed for researchers to choose the most suitable database based on their needs – for example, Reactome^149^ and WikiPathways^150^ are useful for pathway data sharing due to the way the data is formatted and readable in third-party programs. Pathway data are contained in a number of databases (Table S6, ESI†), and include KEGG^151^ (mainly metabolic pathways), Reactome^149^ (manually curated), WikiPathways^150^ (collaborative database), HumanCyc^152^ (mainly metabolic pathways, but also annotated with gene essentiality and other protein features), and Pathway Commons^153^ and BioSystems^154^ (integration and standardisation of several databases). As well as pathway databases, Gene Ontology (GO) annotates biological processes, molecular functions and cellular components with their associated proteins. In GO, rather than being organised as ordered cascades of signalling pathways, annotations can be considered more as a ‘gene set’, organised as a hierarchy and often used in much the same way as pathway data in mechanism of action analysis. GO terms are often considered to be highly redundant^155^ (multiple terms describing the same or similar process), leading to the development of specific tools for “trimming” GO annotations such as REViGO^156^ and GOATOOLS.^157^

**Fig. 3:**
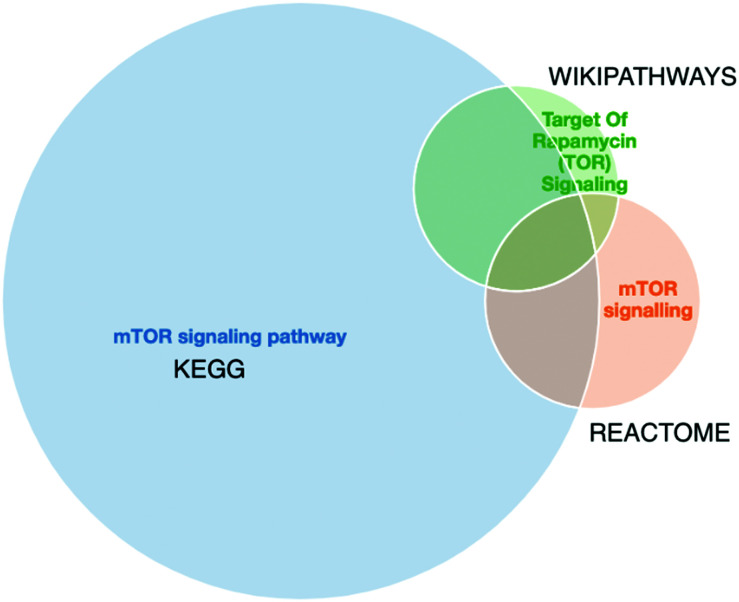
The merged mTOR signalling pathway from KEGG (blue), Reactome (orange) and Wikipathways (green) visualised in PathME viewer. The intersection sizes represent the number of entities in common *vs.* the number of entities in each pathway. We observe that, for the same pathway, the information from 3 different sources varies. Visualisation created with PathMe Viewer.

Furthermore, a final key limitation of pathway databases is the discrepancies found between pathway databases due to differences in data curation. An example of such differences for mTOR signalling pathway from three different data sources is shown in Fig. 3. As we can observe there is no perfect overlap between the three data sources and in this specific case the pathway information retrieved from Reactome is a fraction of the information retrieved from KEGG or Wikipathways. Thus, tools such as PathMe^158^ can be used to interrogate these differences and to extract consensus pathways, or choose the most comprehensive or appropriate annotation database.

## Methods of mechanism of action elucidation

There are a range of methodologies that can be applied to elucidate compound MoA, from network and pathway methods to unsupervised and supervised machine learning. These methodologies differ in their considerations (for example, data required, limitations in annotations, and computational complexity), which we will now further discuss to allow for researchers to choose the appropriate methodology for their particular data type and scientific question. We also provide some implementations of the methodologies in web servers and open-source software packages, as well as helpful supplementary methods to use in tandem to better interpret the results.

### Enrichment methods

#### 1. Connectivity mapping

Connectivity mapping aims to compare a query gene expression signature (gene expression changes in cell lines as a result of treatment with a compound) with a collection of “reference signatures” associated with either a drug/compound with known MoA, or a disease.^58^ This method was popularised following the development of the CMap database, which serves as a repository for reference signatures for this methodology. With connectivity mapping, two signatures can either be positively or negatively connected, *e.g.*, two drugs with high positive connectivity are inferred to share the same MoA, whilst drug signature with negative connectivity to a disease signature can be inferred to “reverse” the disease biology on a transcriptional level. Though an oversimplification of the biological mechanisms of drug treatment, the methodology provides a suitable way to make connections between drug and disease signatures – and by comparing only the top up- and down-regulated genes (where the strongest signal is) the noise in gene expression data is discarded.

**Fig. 4:**
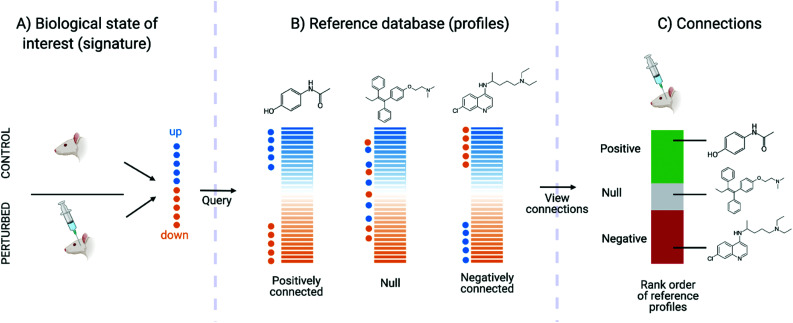
Connectivity map procedure (adapted from original article). (A) The biological state of interest should be represented as a gene expression signature (query), from which the top up- and down-regulated genes are interrogated. (B) The query signature is compared against reference profiles to compute connectivity. (C) The reference profiles are ranked in terms of both magnitude and direction (positive or negative) of connectivity to the query signature.

Connectivity mapping is the matching of compounds to diseases, other compounds or gene knock-out using gene expression signatures. The name comes from the Connectivity Map, which is a set of resources consisting of signatures representing changes in cellular state following systematic molecule, disease, gene, or other form of perturbations and enable characterisation of signatures from novel perturbations based on similarity.^159^ It is carried out with a Kolmogorov–Smirnov (KS)-like nonparametric, rank-based pattern-matching enrichment strategy and results in a “connectivity score” between the query signature (Fig. 4A) and each reference signature. The connectivity score, which ranges from +1 to −1, denotes the extent to which up-regulated query genes tend to appear near the top of each reference signature (ranked by differential expression relative to control) and down-regulated query genes tend to appear near the bottom of each reference signature (“positive connectivity”), or *vice versa* (“negative connectivity”) (Fig. 4B). Each reference perturbagen is then ranked according to their connectivity scores, where those at the top are very strongly correlated to the query signature and those at the bottom are strongly anti-correlated (Fig. 4C). Connectivity mapping has been extensively used to infer the MoA of compounds. For example, CMap proved to be efficient in identifying and generating testable hypotheses about MoA of poorly characterized compounds such as celastrol and gedunin. These compounds were found to be able to suppress the gene expression of androgen receptor (AR) activation in prostate cancer cells based on a high-throughput gene expression-based screen for small molecules.^160^ More practical examples have been outlined by Musa *et al.*^64^ and Trapotsi *et al.*^161^

The benefit of this method is that this is a relatively fast approach, and can be computed using a dedicated online platform (https://clue.io/cmap), making it easy for any scientists to perform this analysis. The results are also very interpretable, as highly connected compounds with known targets or affected signalling pathways can be explored to generate initial hypotheses of compound MoA. The drawbacks of connectivity mapping are that it relies on the comprehensive curation of signatures to query against (which aimed to be addressed by the LINCS project, cataloguing cellular responses for around 30 000 compounds), limiting the approach for compounds with new or undiscovered MoAs. Furthermore, any insight is limited by the completeness of the MoA annotations of reference perturbagens − mentioned in the Introduction, there isn’t a perfect “gold standard” for such annotations as of yet. For example, if a compound is well-connected to a “dopamine receptor agonist” then it is unclear which dopamine receptor is precisely being targeted, and it additionally does not give any pathway-level insight into MoA beyond the target. Additionally, compounds may be annotated with multiple “MoA labels” such as Lisuride, which is annotated as dopamine receptor agonist, prolactin inhibitor, serotonin receptor antagonist, and serotonin receptor ligand^162^ – in this case it would not be clear which MoA label applies to the query compound. One disadvantage is that the connectivity scores can vary widely between actual statistical methods and usually there is uncertainty and ambiguity as to which methods are the best. On the other hand, the impact of those choices depends on the application area – where more subtle changes in gene expression need to be considered, such as in MoA analysis, methodological choices (as well as noise in the data) will play a bigger role. In areas such as repurposing, where only the strongest signal (*e.g.*, the 50 most up- and downregulated genes) can be considered, both methodological choices and noisy data often play a relatively less important role.

As well as the aforementioned web server, Connectivity Mapping can be carried out with R packages such as Connectivity Map^163^ and gCMAP.^164^

#### 2. Pathway enrichment

Pathway enrichment methods require -omics data *e.g.* transcriptomics, phosphoproteomics or proteomics, and pathway annotations, resulting in a list of significance scores representing the association of the expression data with each pathway interrogated. In this way, significantly enriched pathways can be related to a compound's mechanism of action in terms of the biological processes and cascades the compound is hypothesised to perturb. The most valuable aspect of pathway enrichment analysis is that they allow large lists of genes or proteins with no biological context (*e.g.* from transcriptomic, proteomic or phosphoproteomics experiments) to be reduced down to a smaller number of processes, which are inherently more interpretable than gene lists,^165^ and this biological understanding can help to rationalise the phenotypic finding in question.

The hypergeometric test is considered to be the simplest approach to perform pathway analysis and it works by quantifying the overlap between a set of differentially expressed genes (or other features) detected in the high-throughput data and a background set of genes – also termed ORA or overrepresentation analysis.^166^ The background genes are usually the full set of measured genes or the whole human genome. The null hypothesis of this test is that the genes of a pathway are not enriched in the differentially expressed genes. This method provides the advantage of being simple and computationally inexpensive, but it can be biased from the arbitrary cut-off used to define the differentially expressed genes,^167^ usually a *p*-value cut-off of 0.05 and absolute log 2(fold-change) of between 1–2.

GSEA (Gene Set Enrichment Analysis) on the other hand is a functional class scoring (FCS) method with the underlying hypothesis that the genes that are involved in a similar biological process or pathway (grouped into gene sets) are coordinately regulated. Previous benchmarking of FCS methods found that GSEA is a powerful method which is able to detect relevant signalling pathways with a high positive rate.^168^ Unlike ORA, this method does not require a defined set of differentially expressed genes, on the contrary it uses some comparison metric for all measured genes. Genes are ranked according to a metric (*e.g.* differential gene expression significance), and then GSEA aims to identify whether the genes from a set/pathway occur in the top or bottom of the ranked gene list. The null hypothesis of GSEA is that no genes in the expression profile are associated with an observation and occur randomly. A Kolmogorov–Smirnov test is then applied to evaluate the statistical significance of the enrichment. The advantage of GSEA is that it does not require an arbitrary cut-off to define differentially expressed genes and it provides a more in-depth characterization of pathways representative in the data compared with the hyper-geometric test.^167^

However, GSEA and ORA are not able to take into account the topology of the underlying pathways (*i.e.* the interconnections of genes or other biomolecules within the pathways). Therefore topology-based pathway enrichment analysis methods were developed as the latest generation of pathway enrichment methods.^122^ Topology-based methods are similar to FCS methods except they incorporate pathway topology metrics such as number of reactions and position of gene, and compute a “pathway impact factor”.^169^ A limitation of this approach is that true pathway topology is dependent on cellular context and organism, and such differences are usually not represented in pathway databases. In addition, concerns have been expressed in the literature that GSEA does not have a well-defined null hypothesis.^170^ For this reason, other possibly better statistical properties have been proposed such as ROMER^171^ and ROAST.^172^

These various types of pathway enrichment methodologies are incorporated in different web servers and software packages. The Reactome website, PANTHER,^173^ Enrichr^174^ and DAVID,^175^ as well as Cytoscape^176^ (network analysis software) apps such as ClueGO^177^ allow for GUI-based pathway/GO term enrichment. The Reactome website only allows enrichment calculations of Reactome pathways, while PANTHER, DAVID, Enrichr, GSEA and ClueGO allow for additional pathway annotations and GO terms to be enriched. Open-source software packages for programmatic pathway enrichment include R packages such as ReactomePA,^178^ ClusterProfiler^179^ and ToPASeq.^180^ ReactomePA performs pathway enrichment specific to Reactome pathways, but ClusterProfiler and ToPASeq allow for more flexible definition of pathways/gene-sets including user-defined sets, as well as allowing the user to use different enrichment algorithms.

**Fig. 5:**
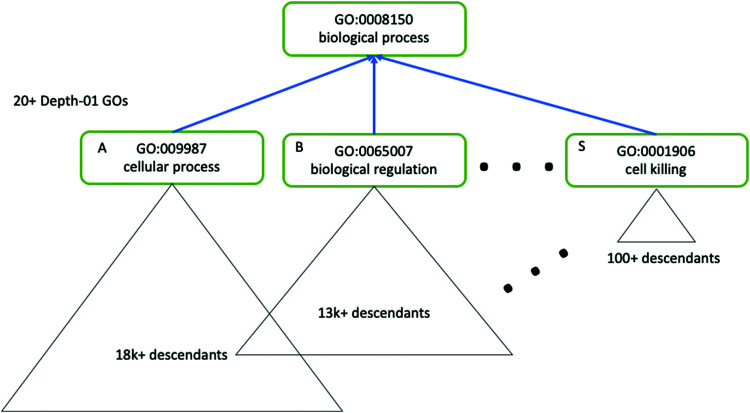
The GO hierarchy is skewed, and contains redundant terms. Tools such as GOATOOLS can be used to correct for the skewed nature of GO ontology. Here, three terms (A, B and S) have the same level of hierarchy but different descendants, which illustrates the complexity of using GO terms for enrichment analysis. Figure adapted from Klopfenstein *et al.*^157^ with permission from the authors, copyright 2018.

As mentioned in *Data and Databases*, GO terms can often be highly redundant, and the hierarchy is skewed such that terms may have different levels of specificity despite falling in the same depth of the hierarchy (Fig. 5). Following enrichment of GO terms the Python package GOATOOLS, the R package GoSemSim^181^ and the web server REVIGO can be useful for easier interpretation of GO terms. Such methods are able to summarise enriched GO terms as a smaller list of informative and non-redundant terms, based on calculated properties of each term such as the Information Content (which uses all GO terms to compute the uniqueness of a particular term), also known as a “semantic similarity” measure.

### Causal reasoning

Causal reasoning refers to a collection of methods that utilises a prior knowledge network (PKN) of signed and directed molecular interactions (*e.g.*, protein–protein) to “reason” upstream from input gene expression data to find nodes in the network which would maximally and accurately explain the observed changes in mRNA expression *via* their known interactions.^182^ When used with compound-perturbed gene expression data, these methodologies infer perturbed nodes or modules from a prior knowledge network in terms of compound-induced modulated signalling proteins, which can then be related to compound MoA. The basic principle of causal reasoning methods is that they view differential gene expression arising as a consequence of perturbed signalling activity; *i.e.*, in contrast to pathway enrichment methods, which equate differentially expressed genes with the signalling activity of their corresponding proteins.^183^ Because transcription factors (and thus transcription and mRNA abundance) are modulated by perturbed signalling arising from *e.g.*, compound-target binding, causal reasoning aims to find, score or optimise the participants of these signalling pathways which have led to the observed (experimentally-measured) changes in mRNA abundance. Thus, they require gene expression data and a prior knowledge network as input and, dependent on the method, output a ranked list of proteins or a signalling subnetwork.

Nodes on a prior knowledge network can be prioritised using a number of methods; for example, by simply counting the number of concordant interactions each node makes with the observed changes in gene expression (CausalR).^182^ Other methods score network nodes by incorporating gene fold-change statistics (SigNet),^184^ or by computing the Kullback–Leibler divergence (relative entropy) of interactions in the network based on the differential expression of each measured gene (DeMAND),^185^ or by using ODE (ordinary differential equation) kinetic approximations of mRNA regulation to estimate the ability of each node on the network to modulate gene regulatory activity (ProTINA).^186^ As well as ranking network nodes, causal reasoning methods can output subnetworks which capture dysregulated signalling cascades (CARNIVAL) – such subnetworks can be optimised using inferred transcription factor activities and pathway weights, and optionally known bioactivity (protein targets),^187^ or from connecting nodes of interest (*e.g.* highly ranked nodes) to input genes *via* their concordant interactions (CausalR).^182^

The choice of algorithm to use depends on the level of understanding of MoA which is required (for example, SigNet, ProTINA and DeMAND are able to recover compound targets whereas CARNIVAL is suited to recovering pathways and modulated signalling proteins). Furthermore, different algorithms are suited to different prior knowledge networks – CARNIVAL has been optimised with the consensus Omnipath network, whereas ProTINA requires a cell-specific network, so this must also be considered when carrying out this kind of analysis.

Causal reasoning is a valuable tool for the understanding of compound MoA as it provides a more biologically correct estimation of perturbed signalling proteins compared to pathway enrichment, as these methods do not falsely equate gene expression with protein activity. In fact, the output from causal reasoning can be used in pathway enrichment methods to understand the biological processes perturbed by the compound in question, and has been found to outperform pathway enrichment on the gene-level for recovering relevant compound target-associated signalling pathways.^187^ These aforementioned methodologies use protein–protein interaction networks with gene expression data as input, but multi-omic approaches have also been developed which perform causal reasoning analysis on several layers (metabolic networks, gene regulatory networks and protein–protein signalling networks) using metabolomics, phosphoproteomics and transcriptomics data.^188^ Owing to the availability of metabolic, gene regulatory and protein–protein interaction networks, these methods allow for intuitive data integration, which likely will become more popular once metabolomic and phosphoproteomic data becomes more available in the public domain.

Overall limitations of causal reasoning approaches are that they can often be quite computationally intensive, especially as network size increases, due to the increased number of interactions which need to be analysed. Additionally, there can often be a connectivity bias if not explicitly corrected for, where nodes which are more connected in the network will be prioritised more often by the algorithms. However, it can be argued that this is not necessarily incorrect, as nodes with more connections are often more well-studied, and thus more crucial to cellular processes. A key limitation of systems biology methods in general is the lack of validation to validate signalling protein inference the output must be compared to experimentally measured protein activity changes, which is generally less available in the public domain along with concurrent transcriptomics data.

The algorithms described above are implemented in open-source R packages including CARNIVAL, CausalR, DeMAND and PROTINA, and SigNet is implemented in the commercial CBDD software.^189^ Additionally, GUI-based causal reasoning can be performed in commercial software such as MetaCore (Key Pathway Adviser)^190^ and Ingenuity Pathway Analysis^191^ with their own bespoke prior knowledge networks.

### Unsupervised machine learning

Unsupervised machine learning (ML) refers to algorithms which use unlabelled data to extract features and patterns, and include methods such as clustering and factor analysis.

#### 1. Clustering

Clustering methods are commonly employed as the first step in data analysis to identify groups of samples that are may be related or interacting.^192^ Therefore, they are preferred as exploratory tools rather than predictive or hypothesis building analyses. Grouping of data into clusters is based on similarity or distance-based metrics (*e.g.*, *k*-means clustering) or based on data density (*e.g.*, DBSCAN). Clustering is usually used to analyse unstructured and high-dimensional data such as gene expression, chemical and image-based data in order to better understand biological processes on various biological levels.^193^ The most popular clustering algorithms are grouped into 3 different categories; hierarchical clustering (HC), centroid-based clustering (CC) and density-based clustering (DB). Moreover, Deep Neural Networks (DNNs) can be efficient in transforming mappings from a high-dimensional data space into a lower-dimensional feature space, which theoretically can lead to improved clustering results and an extensive review on such methods has recently been published by Karim *et al.*^193^

Clustering is relatively fast (in particular centroid-based and density-based clustering, while hierarchical clustering is more time-complex)^194^ and is able to be carried out on one or multiple levels of data, thus clusters can be compared in different spaces. It can also be useful when compounds are annotated with their MoA – if compounds which share the same MoA cluster together in a particular biological space, query compounds can be interrogated for their cluster identity and thus MoA. However, MoA elucidation by clustering has the same limitation as Connectivity Mapping when clusters are compared to ground-truth annotations, where the level of insight you can gain is limited by the annotations (and their associated completeness, coverage and biases). Moreover, Karim *et al.* concluded with deep learning based-clustering that the main consideration when applying such an approach is that there is a lack of labelled data for *e.g.*, gene expression and bioimage data, but NNs require many samples to converge towards generalisation. Hence, they suggested to use transfer learning in combination with this approach.^193^

One major consideration in clustering analysis is the choice of clustering or similarity method, and in a recent comparison of 13 well-known clustering methods, which were applied on 24 biological datasets ranging from gene expression to protein domains, the main conclusion was that there is no universal best performing clustering method.^192^ Results of this analysis were used to develop ClustEval; a publicly available guideline for biomedical clustering tasks, which can be used to choose an appropriate clustering algorithm for the particular scientific question.^195^

A wide range of clustering methods are implemented in Python (scikit-learn^196^) and R (cluster,^197^ factoextra^198^) packages, as well as in online frameworks such as the aforementioned ClustEval.

#### 2. Group factor analysis

The increasing need in MoA studies to explore multiple biological layers in parallel spanning the genome, transcriptome, metabolome, proteome and cell image – space has paved the way for the development of methodologies that can perform integrative analyses.^199^ An example of such approaches is Group Factor Analysis (GFA), which is a dimension reduction technique aiming to explain correlations in a set of data and relate variables to each other.^200^

GFA is a method that can search for relationships between different types of data such as chemical descriptors and biological processes.^201^ GFA captures relationships (statistical dependencies) by explaining a set of data sets (‘views’) by a reduced (low-dimensional) representation called factors or components.^202^ An implementation of GFA developed specifically for factor analysis of multiple types of biological data is Multi-omics Factor Analysis (MOFA), which is proposed as an improvement of previous factor analysis methodologies by enabling analysis of sparse datasets, computational scalability to larger datasets and non-Gaussian data modalities, such as binary readouts.^199^

**Fig. 6:**
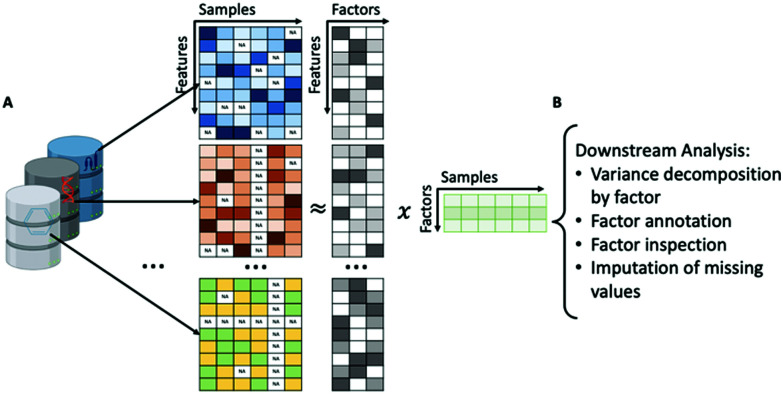
(A) Demonstration of model overview. Multi Omics Factor Analysis (MOFA) takes a number of data matrices as input from different data modalities and decomposes these matrices into a matrix of factors for each sample and weight matrices, one for each data modality. (B) Downstream analysis of MOFA model including variance decomposition, assessing the proportion of variance explained by each factor in each data modality, inspection of factors and imputation of missing values. Created with BioRender.

MOFA, given a set of data modalities, infers interpretable low-dimensional factors (Fig. 6A), using group factor analysis. These factors or components capture the major sources of variation across the data and hence enable the identification of continuous gradients or discrete subgroups within the samples. In addition, MOFA can explore to what extent each factor is unique to a single data modality or is manifested in multiple modalities, revealing shared axes of variation between different omics layers. Once the MOFA model is trained the option for downstream analysis (Fig. 6B) includes visualisation, clustering and classification of samples in factor space.

Group factor analysis methods offer the advantage to integrate multiple data types which enables a data-driven, systems-level analysis of compound MoA, but there are some limitations associated with such methods. Key challenges are the requirement of multiple parameters to be determined, computationally demanding cross validation, manual parameter tuning and prior information may be required for interpretation of results, such as annotations.^203^ In addition, the factors learned from factor analysis can often be difficult to interpret, but methods such as MOFA overcome this limitation through automated annotation of factors using enrichment analysis, and identification of outlier samples.

The MOFA and, more recently, MOFA+ (which is able to deal with single cell data^204^) methodologies have been implemented in both R and Python packages, and general group factor analysis can also be performed with the GFA^205^ and GFAsparse^201^ R packages. There are also other types of methodologies developed for multi-omics data integration based on different approaches such as similarity-, correlation-, network-, Bayesian-multivariate-based. For example, iClusterPlus, which is a Bayesian-based approach uses penalized likelihood approach with lasso penalty to associate a genomic feature with a phenotype. This tool has associated integrated clusters with the pharmacological profiles of 24 anticancer compounds and revealed a selective sensitivity to MEK inhibitors in a subset of haematopoietic cell lines, which is a potentially clinically important finding. For more information on different multi-omics methodologies and their applications, we suggest a detailed review by Subramanian *et al.*^206^

#### 3. Supervised machine learning

Supervised machine learning (ML) methods are applied to train a model and identify patterns when labels are available.^207^ For drug–target prediction models, the labels are usually extracted from bioactivity databases (see bioactivity data section) and are actually experimental evidence of interaction or not between drugs and targets. The labels can be in the form of binary data (*i.e.* a compound to be active or inactive), continuous data (*e.g.* IC_50_ values) or censored data (*i.e.* activity is above or below a threshold). Binary data or binarized continuous or censored labels are used to train classification models, whereas continuous data are used to train regression models. These labelled data are used to optimise a function which is able to connect features (*e.g.*, gene expression or compound structure descriptors) to an endpoint (*e.g.*, the activity of a compound at a particular target; the label). There are numerous supervised machine learning methodologies, which are applied in various stages of the drug discovery pipeline and which can potentially improve discovery and decision making for research questions when data is available.^208^ From the perspective of understanding compound MoA, supervised ML has extensively been used in target prediction of primary drug targets (using bioactivity data as the endpoint modelled)^23^ and also of potential off-target interactions.^209^

Chemical structure information (*e.g.*, binary fingerprints indicating the presence or absence of substructures^210^) has been widely used as features in target prediction tools,^42^ though there are cases where the chemical structure information might not be appropriate or enough to inform of a compound's bioactivity or response to biochemical assays. An example of such a case is the presence of ‘Activity Cliffs’, where only small transformations to similar structure compounds result in a large difference in potency and bioactivity profiles.^211^ Indeed, it has been shown that only 30% of compounds with high similarity to an active compound are themselves active at the same target.^212^ This highlights the need for additional compound representation beyond chemical structure. Examples of such descriptors are the expression response of the 978 LINCS “landmark genes”^213^ or cell morphology changes in the form of microscopy images or calculated features.^214^

After the selection of appropriate compound features, supervised ML is carried out by training a model (fitting a function linking the descriptors to the end-point) and then testing it on a held-out test set to understand how well the model performs with new ‘unseen’ data, with an optional validation set used to optimise various hyperparameters of the models. Cross-validation (CV) is a useful strategy for smaller data sets, as it splits the data into ‘*k*’ folds (where *k* is the number of folds defined *a priori*) which are subsequently split into multiple training and test sets. There are various methods to split the data into *k*-folds; for example, a stratified split is used to preserve the percentage of samples for each class in each fold or a group-based split is applied to group compounds based on a property/characteristic (*e.g.*, chemical scaffold) and compounds with the same characteristic will either be present in the train or test set in each fold. It must be kept in mind, however, that different types of split strategies give very different results. For example, in a comparative study between different CV methodologies, the scaffold (group)-based CV was found to be pessimistic, the random selection of compounds in train and test set was overoptimistic and the time series split in addition to random selection was suggested as a most realistic CV approach.^215^

There are a variety of algorithms that can be used to train models. Random Forest (RF) methods build an ensemble of decision trees based on the features which are better able to classify the data. Support Vector Machines (SVM) represent each data point in *n*-dimensional space (where *n* is the number of features), and a hyperplane is found in this space which differentiates the classes or labels. RF and SVM are usually used in a single-task setting; *i.e.*, in the case of target prediction, one model has to be built for each target and thus for a query compound multiple models need to be applied to understand which targets it is potentially active against. In fact, target prediction models can learn from each other to improve classification accuracy.^216^ Bayesian Matrix Factorisation or BMF is a machine learning algorithm which learns multiple tasks (such as predicting multiple drug targets) simultaneously, and the learning tasks can then benefit from each other. The approach works by factorising a sparse matrix *Y* (*N* compounds times *M* targets) containing compound bioactivities to a lower-dimensional representation in latent matrices u and v, for compounds and targets respectively.^217^ With BMF it is possible to integrate multiple data types by incorporating side information (such as transcriptomic or cell image features).^218^

More recently, deep learning (DL) methodologies have attracted more attention for their ability to learn representations of data with multiple levels of abstraction and also their good performance.^219^ DL methods are a type of Artificial Neural Network (ANN) with multiple hidden layers in combination with more sophisticated training parameters, which aim to emulate the complex neuronal system (and the process of learning) in the human brain. Specifically, Deep Neural Networks (DNNs) refer to ANNs with many hidden layers, and Convolutional Neural Networks (CNNs) are ANNs which have a convolution layer and a pooling layer (and have shown to be beneficial for processing image data^220^). CNNs in particular can also be used to automatically extract features from cell morphology data^30^ for use in further modelling or unsupervised ML approaches such as clustering.

The choice of which method to use for bioactivity prediction is not entirely clear and is hence still an area of active research. Different methods have been compared for their ability to predict compound targets, in particular the performance of approaches such as RF and SVM have been compared to NNs. Mayr *et al.* published a seminal benchmarking study using bioactivity data from ChEMBL which found that deep learning methods outperform other methods (RF and SVM, as well as *k*-Nearest Neighbour and Naive Bayes predictors), and are close to the accuracy of *in vitro* wet lab experiments, based on the AUROC (area under receiver-operating curve, true positive rate/false positive rate) metric.^221^ In response to this, Robinson *et al.* performed the study again, this time questioning the usefulness of the AUROC metric for bioactivity prediction and thus also assessing the area under precision–recall curves (AUPRC), which is useful when using imbalanced datasets (*i.e.*, many inactives to a handful of actives, commonly seen in bioactivity data). This study concluded that SVM in fact performs comparably with deep learning methods, in terms of the AUPRC.^222^ This highlights the fact that model evaluation is often difficult and has been reviewed previously, with the conclusion that evaluating a model is virtually practically impossible and thus comparing models is not a trivial task.^223^ In addition, a comparison between BMF and RF methods for predicting bioactivity was also undertaken, finding that they performed similarly when compound structure features (ECFP fingerprints) were used, but interestingly BMF outperformed RF for the majority of target classes when cell morphology-based features were used, thus the choice of which feature to use to represent compounds is important when deciding on a supervised ML methodology.^19^ It can thus be concluded that how well a method appears to perform depends heavily on the end-point being modelled, the data going into the model and the evaluation metric being considered. Therefore, it is generally difficult to know *a priori* what is the best combination of supervised ML methods and compounds’ descriptors. However, experience has shown that imaging data benefit strongly from deep learning such as Convolutional Neural Networks, whereas tabular data such as molecular data or image-based features less so.^224^ Despite the advantages of using CNNs, it is important to keep in mind that their performance could be limited by the data availability as often imaging datasets are small and heavily conditional.^225^ In fact, other important model characteristics such as the applicability domain (where the model works with high reliability and where it doesn’t, for example in terms of areas of new chemical space, *e.g.*, Reliability Density Neighbourhoods^226^) and prediction uncertainty (Venn-Abers, conformal prediction)^227,228^ should also be considered, as well as performance-based measures such as accuracy, AUROC and AUPRC, but are often neglected in bioactivity model evaluations despite providing a measure of how confident one can be in new predictions (which is the ultimate goal of target prediction, and any supervised ML model).

In general, the benefits of unsupervised ML for mechanism of action understanding (particularly for target prediction) are that they are able to be trained with any kind of data including the -omics data discussed in this review, and that they achieve high performance for predicting targets, which was found in the comparison studies discussed above. Drawbacks of supervised ML are the need for data coverage in both chemical and endpoint space, the potential for overfitting (high accuracy on training data but poor generalization to new data, often overcome with feature selection,^229^ and early stopping in NNs^230^), and computational time particularly using deep learning methods which often require access to GPUs.^231^ Futhermore, machine learning approaches have been likened to a “black-box”, where data goes in, predictions come out, and what happens in-between is unclear.^232^ Feature importance calculations in Random Forest models can somewhat overcome this limitation, as it is possible to understand which features are most effective at classifying samples – and if the features are genes and proteins then these can be further biologically interpreted.^233^ Interpretable deep learning methods have also been developed to address this limitation, including “knowledge-primed” neural networks where protein–protein interactions are used as the network architecture, hence node activations during model training can be related directly back to mechanistic activity.^234^ The current case studies have focused on understanding cellular regulation in transcriptomics data capturing disease states such as leukaemia, but it could in the future be applied to compound-perturbed transcriptomics data to understand the cellular responses to compound administration as a novel application.

Many algorithms such as RF and SVM can be carried out with scikit-learn functions in Python,^196^ deep learning with TensorFlow^235^ and PyTorch,^236^ and BMF with ‘macau’^237^ and ‘smurff’^218^ python packages. Some implementations of these methodologies for understanding compound MoA include PIDGIN, a target prediction tool, which uses positive (‘active’) data from ChEMBL and negative (‘inactive’) data from PubChem to build a collection of classification RF models.^42,238^ We have recently released the 4th version of PIDGIN, which includes data from ChEMBL 26 and PubChem (extracted in March 2020) and can been accessed from Bender group github page (https://github.com/BenderGroup/PIDGINv4) and documentation is also available (https://pidginv4.readthedocs.io/en/latest/).

We have summarised the tools and software that are mentioned in this section in Table S7 (ESI†).

## Applying the methods to the data: case studies

As we highlighted in the previous sections, there are many methods and data types that can be used alone or in combination to better understand the challenging concept of MoA. Each method, despite its advantages, also has limitations associated with it; for example, network and pathway methods rely on the curation quality of the prior knowledge, ML can be time consuming and results difficult to interpret, and potential insights gained through connectivity mapping are restricted to a small part of high-level MoA space. Similarly, to methods, different data types capture a different part of the MoA biology and thus enable a more comprehensive understanding of compound MoA. In this section and in Table 3, we will (1) summarise some case studies on MoA elucidation using a variety of methods, (2) review approaches that use different types of data or integrate multiple omics-data and (3) highlight the utility of lesser-available data types (*e.g.*, proteomics). We selected the following case studies to include the full range of data types and methodologies outlined in this review, in particular where data or methods were integrated to gain complementary information on compound MoA.

**Table 3:** Applications of different methods and data modalities to gain understanding of compound MoA

Application type	Data type(s)	Method(s)	Scientific findings	General learnings	Ref.
Integration of data	Phosphoproteomics	Causal	Generated detailed mechanistic hypotheses, *e.g.*, for Trichostatin A and MS-275 (HDAC inhibitors) inhibiting the downstream HDAC1 pathway and causing cell growth arrest *via* activation of p53 and p21	Phosphoproteomics data was used to enhance network inference using transcriptomics data, but the approach was limited by data availability	Ji *et al.*^239^
	Transcriptomics	Reasoning			
	Network				
	Pathway				
	Proteomics	Pathway enrichment	Proteomics analysis showed specific compound-induced increases and decreases on the protein expression level of proteins relevant to cytoskeletal regulation and signal transduction pathways in neurons, and were related to the changes on the mRNA level to hypothesise the signalling cascades modulated by the compound	Pathway enrichment analysis on a set of proteins/genes derived from proteomics and transcriptomics data of a compound can put the genes/proteins into biological context and further better understand a compound's mechanism of action	Weinreb *et al.*^240^
	Transcriptomics				
	Pathway				
	Transcriptomics	Machine learning	Each type of molecular data was mapped to a network of molecular interactions	Machine learning network models on multiple -omics spaces are able to prioritize disease-relevant mechanisms of action	Patel-Murray *et al.*^241^
	Proteomics		Network optimization of this large interactome highlighted the functional changes induced by the compounds		
	Metabolomics				
	Epigenomics				
	Cell image	Machine learning	Cell image data used in bioassay prediction increased hit rates of two internal Janssen projects and hits were chemically diverse	Cell image data can be useful and, in some cases, complementary to chemical structural information for bioactivity predictions	Simm *et al.*^242^
	Bioactivity				
	Cell images derived from cell types treated with:	Deep learning	Immune signalling modelled with images of cells and be used for the development of accurate disease models, which proved to facilitate the discovery and MoA understanding of immune modulating drugs	Cell Painting data derived from different types of treatment can be used to develop disease models and identify potential treatments, at the same time understanding their MoA	Cuccarese *et al.*^243^
	(a) Recombinant proteins		Methodology applied on the context of COVID-19 and identified drugs currently in clinical trials for COVID-19		
	(b) CRISPR-based genetic modifications				
	(c) Small molecules				
Integration of methods	Transcriptomics	Connectivity mapping	Application of two methodologies enabled the researchers to generate the novel NF-κB hypotheses for the MoA of pinosylvin	Two complementary methodologies were applied to generate novel hypotheses for the MoA of anti-inflammatory compound pinosylvin, similar mechanisms suggested by two separate methods increasing confidence in the hypothesis	Kibble *et al.*^244^
		Group factor analysis			
Integration of data and methods	Cell image	Pathway enrichment	Functional enrichment analysis on Nomilin, Zardaverine and Hydrocotarnine identified genes involved in the regulation of cytoskeletal remodelling and growth activation, hence cellular changes in the cytoskeleton in addition to its role in determining cell morphology produce changes in gene expression	Significant associations between alterations in cell morphology and gene expression were identified	Nassiri and McCall^32^
	Transcriptomics	Machine learning		A set of genes associated with an image-based feature and resulted in a better understanding of the biological responses to compound perturbations	
	Pathway				
	Transcriptomics	Machine learning	Phenothiazine was predicted to interact with the androgen receptor (AR) based on its high transcriptional similarity with enzalutamide (despite low chemical similarity), which is indicated for prostate cancer	The combination of transcriptional similarity with pathway enrichment analysis provided new (and experimentally validated) therapeutic indications for compounds across different diseases, meanwhile chemical similarity alone would not have led to this hypothesis	Iwata *et al.*^245^
	Bioactivity	Pathway enrichment	An *in vitro* cellular assay validated that phenothiazine inhibits AR		
	Pathway				
	Proteomics	Clustering	Pathway enrichment analysis on biopsies identified factors (proteins and phosphosites) that are up-regulated specifically in hepatocellular carcinoma upon sorafenib treatment	Proteomics and phosphoproteomics data from biopsies can contribute to precision medicine based on phenotypic data to identify new targets, biomarkers and signalling pathways that mediate drug resistance	Dazert *et al.*^246^
	Phosphoproteomics	Pathway enrichment			

### Integration of data

Because causal reasoning approaches generate hypotheses for modulated signalling proteins, phosphoproteomics data (which measures changes in protein signalling) can be integrated with transcriptomics data as a complementary source of data for this methodology. A causal reasoning implementation was developed by Ji *et al.* wherein cell-specific pathways for a set of compounds were elucidated by integrating both gene expression and phosphoproteomics data in a binary linear programming (BLP) implementation to infer drug targets from prior knowledge networks.^239^ However, they were severely limited by the data availability (15 compounds with both gene expression and phosphorylation data in the LINCS public repository). We expect that the use of this type of data will be improved in the future due to efforts for increasing data deposition in the P100 repository.^41^ In conclusion, with this method they were able to generate detailed mechanistic hypotheses, such as Trichostatin A inhibiting the HDAC1 pathway and causing cell growth arrest *via* activation of p53 and p21, thus highlighting that the combination of transcriptomics and proteomics data is useful in understanding of a compound's effects on the pathway level and thus its mechanism of action.

A combination of transcriptomics and proteomics data was also used by Weinreb *et al.* to demonstrate an effective way to integrate transcriptomic and proteomic data for understanding the MoA of Antioxidant-iron chelator green tea polyphenol (−)-epigallocatechin-3-gallate (EGCG) and to further rationalise its neurorescue impact in aging and neurodegenerative disease.^240^ They performed pathway enrichment analysis on both data modalities, showing differences in expression from the proteomic analysis and differential expression levels from the transcriptomics analysis. By viewing the data on both the gene and protein-levels, mechanistic insights were gained such as the finding that EGCG reduced the protein and mRNA expression levels of a key enzyme which negatively regulates the stability and degradation of several proteins involved in cell survival and differentiation. Overall, the study succeeded in generating a list of proteins and genes from two different -omics spaces (proteomics and metabolomics) which were related to various biological pathways underlying EGCG's neuroprotective mechanism of action.

The similarity of query compounds to reference compounds has been extensively used as a strategy to better understand MoA with the main limitation that it is limited by the number of compounds with known MoAs and ‘gold standard’ annotations. Hence, Patel-Murray *et al.* proposed a multi-omics (transcriptomics, proteomics and metabolomics, as well as epigenomics) approach which does not require reference compounds or large databases of experimental data in related systems, and thus can be applied to the study of agents with uncharacterized MoAs and to rare or understudied diseases.^241^ To understand the MoA of a set of compounds in Huntington's Disease (HD), they clustered the data in each -omics space. In gene expression space, the profiles formed only one distinct group, whereas two distinct groups were observed in the metabolite profiling data. Interestingly, the compounds clustered together did not have the most similar chemical structures This observation highlights that the assumption of “compounds’ with similar profiles should share similar properties” is not always true and it depends on the type of -omics data used, and the level of biology. To reveal the MoAs for the compounds in the clusters, they applied an interpretable ML algorithm, which mapped each data modality to a network of molecular interactions. In conclusion, they identified and subsequently experimentally validated HD MoAs and thus we observe the value of an approach that combines multi-omics with an interpretable ML method to determine previously unknown MoAs, even in the absence of a comparable reference.

Going beyond transcriptomics, proteomics and metabolomics data, cell morphology information derived from cell images have been used for bioassay prediction in a large scale study, which focused on the repurposing of proprietary image-based data (comprising 500 000 compound treatments) for biological assay prediction.^242^ They aimed to investigate (a) whether image data could overcome limitations employed by chemical descriptors and (b) if image data can be complementary to the chemistry-based models for the sparse and poorly annotated chemical space. Two multitask prediction methods were used, namely BMF Macau and Deep Neural Networks (DNNs). Both methods proved to successfully predict bioactivity using image-based data, performing with an overall AUC-ROC of 0.65 and 0.67 across 535 assays for BMF Macau and DNN respectively. Image-based features were next applied to two discovery projects during virtual screening, increasing the base hit-rate from 50- to 250-fold over that of the chemical structure-based models. Therefore, image-based data proved to be a rich source of information that can be used to predict the result of biological assays, and hence also for MoA elucidation – proving to also be complementary with traditionally-used chemical features in areas of sparse chemical space.

Cell painting data are not only used as features for target prediction, but have also been used in a more novel way to develop disease models and identify potential treatments. In a recent study with the aim to identify immune-modulating drugs, Cuccarese *et al.* developed a ‘phenomics’ platform of fluorescence microscopy images to examine cellular responses to a wide range of perturbations,^243^ namely recombinant proteins, CRISPR-based genetic modifications and small molecules. Deep learning featurisation of cellular images, or “phenoprints”, was performed with CNNs. Firstly, they evaluated whether the phenoprints could capture known functional relationships across a diverse range of immune functions. They showed that the immune signalling repertoire can be modelled with images of cells, and hence can be used with confidence for the development of accurate disease models. These models further facilitated the discovery and MoA understanding of immune modulating drugs. They selected two immune phenoprints (TGF-β- and TNF-α-induced) and screened 90 000 chemically diverse compounds on their phenomics platform, discovering a novel compound able to ‘reverse’ the immune phenoprints at a low concentration. In addition, they demonstrated that drugs in clinical trials for COVID-19 (such as remdesivir) modulated disease models developed with their phenomics platform. Therefore, the development and use of disease models using cellular images derived from multiple types of perturbations integrated in a single phenomics platform can provide information about compounds that modulate them, and as a result better understand their mechanism of action.

### Integration of methods

Equally important to the integration of different data types is also the integration of different methods with the aim to leverage the advantages of multiple methodologies in MoA understanding. For example, Kibble *et al.* generated microarray data for pinosylvin (a natural product which shows anti-inflammatory effects) in two cell lines utilising both enrichment methods (MANTRA or Mode of Action by Network Analysis,^247^ a method similar to connectivity mapping) and unsupervised machine learning (GFA) to obtain a network pharmacology view of the compound's MoA.^244^ They also utilised bioactivity and pathway data to increase their mechanistic understanding. Using bioactivity data, the authors extracted the known targets of the closest connected neighbours to pinosylvin and then queried Pathway Commons for common pathways containing each target. By supplementing the bioactivity data with pathway data, they found that all nearest neighbours except for one mapped to NF-κB pathway inhibition downstream of EGFR. To add to their hypotheses, they utilised the GFA unsupervised machine learning method; decomposing the transcriptomics data derived from pinosylvin and the CMap compounds into factors or ‘components’ in a data-driven fashion. Notably, one component captured HDAC inhibitors, which can reprogram NF-κB response in cancer cells. In this way, they increased their confidence in the NF-κB hypothesis of pinosylvin action by obtaining the same hypothesis with two distinct methodologies.

### Integration of methods and data

Transcriptomics data can also be effectively integrated with cell image data, as changes in cell morphology and gene expression both reflect changes in activity in effector proteins following a perturbation in signalling, where it is not known in detail how these processes interact. Nassiri and McCall developed a pipeline for linking the two types of data together and integrating them for MoA understanding.^32^ They utilised the LINCS gene expression dataset as well as the Broad cell morphological image collection to extract a set of 9515 drugs and small compounds with data on both levels, their ‘reference database’. They used the reference database to identify compounds with similar gene expression changes, followed by ‘cell morphology enrichment analysis’, which involves the identification of significant associations between alterations in cell morphology and gene expression. ML was then used to model the association between each image-based feature and the landmark genes. The enrichment and modelling methods produced a set of genes (with similar expression patterns) associated with each image-based feature. They demonstrated the pipeline on three compounds and were able to better understand the regulatory mechanisms linking the changes on the gene expression and cell morphology levels induced by the compound by performing pathway enrichment with the query-specific cell morphological gene sets. This study revealed a novel interdependence between gene expression and cell morphology and proposed a method to interpret this in terms of compound mechanism of action through the integration of data and methods. The significance of this finding as the authors concluded is that “We anticipate the results of this study will […] provide a blueprint for the integrative analysis of other multi-omics data, such as mass spectrometry-based targeted proteomics (LINCS P100)”.

Transcriptomics data can also be used with bioactivity data to predict novel compound targets, with pathway enrichment providing further mechanistic insight beyond target engagement. Iwata *et al.* identified active pathways, target proteins and therapeutic indications for ∼16 000 small molecules in 68 human cell lines.^245^ Their pipeline involved identifying active pathways through pathway enrichment of top up- and down-regulated genes, predicting potential target proteins based on transcriptional similarity and bioactivity data, and finally using the predictions to generate an interactome of compounds, target and diseases for the purpose of discovering new therapeutic indicates. For example, phenothiazine was predicted to interact with the prostate cancer-relevant androgen receptor (AR) based on its high transcriptional similarity with enzalutamide (despite sharing a low chemical similarity), which is already indicated for prostate cancer. Moreover, from the pathway enrichment analysis, the apoptosis pathway was detected for both enzalutamide and phenothiazine. An *in vitro* cellular assay experimentally validated the prediction that phenothiazine inhibits AR. In conclusion, this integration of methodologies and data proved to be efficient in the understanding of MoA and compound repositioning and shows how the use of transcriptional similarity in combination with pathway enrichment analysis provided new therapeutic indications for compounds across different diseases.

The integration of data and methods to understand compound MoA can also be used to facilitate precision medicine approaches. In a study using proteomics data conducted by Dazert *et al.*, proteomics data were used in combination with phosphoproteomics data and pathway data with the ultimate aim to enhance our understanding in precision medicine for hepatocellular carcinoma (HCC).^246^ The authors aimed to understand whether the two complementary data modalities could reveal signalling pathway activity in a tumour sample, and understand mechanisms of tumour resistance to sorafenib therapy. Two methodologies were applied to gain insights into the data; hierarchical clustering (unsupervised ML) followed by an enrichment analysis. Hierarchical clustering identified factors (proteins and phosphosites) that were up-regulated specifically in the tumour upon sorafenib treatment. Pathway enrichment analysis on these factors revealed several pathways biologically relevant to the MoA of the compound. To further understand potential mechanisms of sorafenib resistance, they compared pre- and post-treatment tumour biopsies using pathway enrichment on the two data modalities. Their analyses revealed significant enrichment of cell adhesion pathways, which are possible processes involved in tumour progression and sorafenib treatment failure. Therefore, this proof-of-concept study showed that using quantitative proteomics and phosphoproteomics data from biopsies with unsupervised ML and pathway enrichment can contribute to precision medicine based on phenotypic data to identify new targets, biomarkers and signalling pathways that mediate evasive resistance.

Overall, this small selection of case studies (Table 3) illustrates that generally greater availability and integration of different -omics data and the use of multiple complementary approaches/methods can help to overcome limitations specific to the data used, and of each methodology applied, hence supporting the statements made in the introduction that the mechanism of action of a compound needs to be considered from multiple angles in parallel.

## Conclusions and future directions

In this review we aimed to give an overview of the different levels of data that compounds’ MoA can be described on, the data representing these levels and their availability in databases, the methods that can be applied to generate hypotheses from this data, as well as our opinion on their value to the field and avenues for integration. We also highlighted some interesting case studies which effectively applied and integrated different methodologies and data types for understanding the mechanism of action of a particular compound or compounds.

The main aspect which we hypothesise will give the greatest improvement to the field is increasing the availability of multi-omics public data which catalogues the cellular response to compound perturbation on, for example, the phosphoproteome and metabolome level as well as the transcriptome. This view is shared by other members of the scientific community, who note that this is a challenging task due to the complexity of data storage, quality control and compliance with FAIR principles when dealing with multi-dimensional data.^248^ We have observed how open source transcriptomic and cell-image databases have enabled not only the ability to develop more sophisticated methodologies to exploit the data, but to also improve MoA understanding by enabling a more comprehensive reference database for methods such as pattern matching, machine learning and clustering. Moreover, we hypothesise that the data resolution and dimensionality will also increase by including more cell lines, perturbation times and doses in the databases. We expect that a similar initiative for other -omics data would have the same effect on the field, especially with regards to the development of multi-omics methods for understanding mechanism of action on a deeper level – methods for data integration will only become more commonplace if the data is made publicly available.

Another way to improve the field of MoA elucidation could be addressing the curation bias of pathway and network data, which is a valuable source of supplementary information to contextualise and interpret -omics data but is dominated by cancer-related proteins and processes. We also anticipate an increase in “interpretable deep learning”, such as the knowledge-primed neural networks. The developments in the field of Deep Learning have significantly contributed to the field; nevertheless, we believe that these methods should ideally be interpretable for computational researchers to be able to properly rationalise and communicate their predictions to biologists and other bench scientists.

We hope that this review will give insight to researchers who are in the field of mode of action elucidation and inform them of the best methods and data to use for their own scientific question.

## Author contributions

M.-A. T. and L. H.-G. contributed equally to the research and writing of the review. AB edited and approved the final version of the manuscript.

## Conflicts of interest

There are no conflicts to declare.

## Supplementary Material

CB-003-D1CB00069A-s001
