# Supplementary material for: Computational analyses of mechanism of action (MoA): data, methods and integration
Source: RSC Chem Biol. 2021 Dec 22;3(2):170–200. doi: 10.1039/d1cb00069a (PMC8827085; doi:10.1039/d1cb00069a)
Supplement: CB-003-D1CB00069A-s001 [file CB-003-D1CB00069A-s001.pdf]

Electronic Supplementary Material (ESI) for RSC Chemical Biology.
This journal is © The Royal Society of Chemistry 2021

Computational Analysis of Mechanism of Action (MoA): Data, Methods and Integration

Supplementary Tables of Useful Databases and Resources

Supplementary Table 1: Main sources of bioactivity data, their size/coverage and any additional comments

Source	Size/Coverage<br>As of July 2020	Comments
ChEMBL[1]	2M compounds<br>1.2M assays<br>13K targets	Extraction of compound, assay and bioactivity information from journal articles is performed manually by curators
PubChem[2]	103M compounds<br>253M substances<br>1.1M assays<br>95K proteins	Data provided by more than 350 contributors including university labs, government agencies, and pharma companies. Data include siRNAs, miRNAs, carbohydrates, lipids, peptides, and other substances
DrugBank[3]	13.5K drugs<br>5K proteins	Contains data about FDA-approved drugs as well as experimental drugs going through the FDA approval process
ExCAPE[4]	~1M compounds<br>1,667 targets	Over 70 million SAR data points extracted from PubChem and ChEMBL and merged in one database across 3 species (human, rat and mouse).
BindingDB[5]	312,146 compounds<br>1,858 targets<br>5,928 assays	Database for binding measurements, focusing on the interactions of protein considered to be drug-targets with small, drug-like molecules.

Supplementary Table 2: Main sources of gene expression data, their size/coverage and any additional comments

Source	Size/Coverage<br>As of July 2020	Comments
CMap[6]	More than 7,000 expression profiles representing 1,309 compounds in two different cell lines	The original database to accompany the "Connectivity Map" approach, no longer updated
LINCS[7]	1.3M profiles, 476,251 signatures, 27,927 perturbagens (19,811 small molecule and 7,494 genetic), 9 core cell lines and 77 other cell lines	Scale-up of CMap with data measured on the high-throughput L1000 platform, mainly chemical-induced signatures, also but includes genetic perturbations e.g. shRNA knockdown
GEO[8]	3,735,866 distinct samples (including technical replicates), 61,069 microarray experiment series, 38,696 RNA-Seq experiment series	Researcher-uploaded compilation of gene expression experiments from a variety of platforms (RNA-Seq, microarray), biological systems, and including all types of perturbants e.g. disease
ArrayExpress[9]	73,612 experiments<br>2,493,509 assays	Repository for reproducible and well-documented microarray and RNA-Seq data deposition(ref), covering both chemical and disease perturbants
DrugMatrix[10]	Over 600 compounds measured at different doses *in vivo* in rat liver, kidney, thigh muscle, heart, bone marrow, spleen, intestine and brain, and *in vitro* (primary rat hepatocytes)	Contains gene expression data from highly controlled and standardized toxicological experiments with therapeutic, industrial, and environmental chemicals at both non-toxic and toxic doses (ref) using the Affymetrix microarray and CodeLink technologies (now archived)

Open TG-GATEs[11]	170 compounds both *in vivo* (rat kidney and liver) and *in vitro* (rat and human primary cultured hepatocytes) at various single and repeat doses	Gene expression data from primary cultured hepatocytes of rats and humans following exposure to 170 compounds (pharmaceutical products, etc.), no longer updated (ref)

Supplementary Table 3: Main sources of cell image data and any additional comments

Database/Source	Datasets	Coverage
Broad Bioimage Benchmark Collection (BBBC)[12]	Cell Painting	1,600 compounds
GigaScience DB[13]	Cell Painting	30,000 scale up of the Cell Painting dataset in BBBC
To Be Released	Cell Painting	140,000+ small molecules and genetic perturbations
IDR[14]	Pharmacogenetic Phenome Compendium (PGPC)	Gene–drug interactions for more than 1,200 pharmacologically active compounds by high-throughput imaging<br>(300,000 drug–gene–phenotype interactions in total)
IDR[14]	Idr0088<br>(To be released by Janssen)	1,008 approved drugs manually annotated to 218 unique MoAs<br>Each compound profled at four concentrations in live-cell, high-content imaging screens against a panel of 15 reporter cell lines
Recursion[15]	RxRx19a	1,672 small molecules at 6+ concentrations<br>Three viral conditions (active virus, irradiated, mock)
Recursion[15]	RxRx19b	1,856 small molecules at 4-6 concentrations in three COVID-19-associated cytokine storm conditions

Supplementary Table 4: Main sources of proteomics, metabolomics and phosphoproteomics data, their size/coverage and any additional comments

Type	Source	Size Coverage	Comments
Proteomics	PRIDE (Project PXD009775)[16]	56 drugs in 3 cell lines	Proteome signature library of anticancer molecules
	PRIDE (Project identifiers PXD018569, PXD018570, PXD018571, PXD018572, PXD018573, PXD018574)[16]	53 drugs in 5 cell lines	280 compound-cell line pairs and more than 1,000 proteomes across 5 cell lines
	ProTargetMiner (GSK)[17]	287 A549 adenocarcinoma proteomes affected by 56 compounds	ProTargetMiner serves as a chemical proteomics resource for the cancer research community, and can become a valuable tool in drug discovery.

Metabolomics	MetaboLights[18]	715 studies, 212 of which are in homo sapiens	Contains a wide range of metabolomics datasets in different model systems, as well as supplementary data for spectral annotation, not focused specifically on compound mechanism of action
	EcoPresMet[19]	1,279 compounds in E. Coli	E. Coli only
	Fuhrer et al[20]	> 3,800 gene deletions in E. Coli	E. Coli only
Phosphoproteomics	P100 (Broad Institute)[21]	90 drugs in 6 cell lines	Extends Connectivity Map concept to proteomics and enables recognition of cell type specific activities and therapeutic opportunities

Supplementary Table 5: Main freely available sources of network data, their size/coverage and any additional comments

Source	Size/Coverage<br>As of July 2020	Comments
Omnipath[22]	Human signed and directed protein-protein interaction network:<br><br>7,294 proteins<br>72,190 edges	Includes manually curated, high-confidence data from different sources and as well as protein-protein interactions incorporates transcriptional regulation, ligand-receptor interactions, pathways, and more
STRING[23]	24.6 mil proteins (19,257 human proteins)<br><br>5090 organisms<br><br>>2000 mil interactions	Interactions are given a confidence score based on the evidence; includes computational predictions and homology interactions
BioGRID[24]	700,000+ human physical interactions<br><br>25,000+ unique human proteins<br><br>Human data compiled from 32,000+ publications	Biomedical interaction repository with data compiled through comprehensive curation efforts , including chemical interactions and post-translational modifications
BioPlex[25]	120,000 human protein-protein interactions<br><br>15,000 proteins	All interactions derived experimentally from AP-MS measurements in human cells, with proteins annotated with their subcellular localization, biological function and disease association
ConsensusPathDB[26]	660,318 human interactions<br><br>Of which 448,725 are protein-protein interactions and 165,866 are drug-target interactions<br><br>170,000+ unique entities (genes, proteins, chemicals)	Integrates interaction networks including binary and complex protein-protein, genetic, metabolic, signalling, gene regulatory and drug-target interactions, as well as biochemical pathways. Data originate from currently 32 public resources for interactions, and curated literature interactions
GIANT[27]	1540 genome-scale datasets, encompassing ~61,000 conditions from ~25,000 publications	Leverages a tissue-specific gold standard to automatically up-weight datasets relevant to a tissue from a large data compendium of diverse tissues and cell-types. The resulting functional networks accurately capture tissue-specific functional interactions

HPRD (Human Protein Reference Database)[28]	30,047 proteins<br><br>41,327 protein-protein interactions<br><br>93,710 post-translational modifications	Integrates information pertaining to domain architecture, post-translational modifications, interaction networks and disease association for each protein in the human proteome. All the information in HPRD has been manually extracted from the literature by expert biologists who read, interpret and analyse the published data
IntAct[29]	118,345 interactors and 721,618 interactions (molecular interactions including e.g. drug-gene)<br><br>71,071 experiments and 21,836 publications	Includes interactions between genes, proteins, RNA and chemicals. Interactions are derived from literature curation or direct user submissions and are freely available
BioSnap[30]	Physical PPI:<br>21,557 nodes<br>342,353 edges<br><br>Drug-target network:<br>3,932 nodes (284 drugs and 3,648 proteins)<br>18,690 edges	Contains multiple types of networks encompassing different entities and relationships, as well as tissue-specific networks

Supplementary Table 6: Main freely available sources of pathway data, their size/coverage and any additional comments

**Source**	**Size/Coverage As of July 2020**	**Comments**
Reactome[31]	2,423 human pathways<br>13,248 reactions<br>10,923 proteins<br>1,869 small molecules	Reactome is manually curated and peer-reviewed, pathways are arranged in hierarchy under 27 high-level headings such as “Cell Cycle” and “Metabolism”
KEGG[32]	537 human pathways<br>11,274 drugs	Mainly metabolic pathways, but also contains signal transduction and disease pathways
WikiPathways[33]	1,185 human pathways	Open and collaborative platform for curation of pathways by the biology community
GO[34]	28,923 Biological Processes (BP)<br>11,136 Molecular Functions (MF)<br>4,185 Cellular Processes (CC), across 4,643 species	Not strictly pathways but processes, follows ontology
NCBI BioSystems[35]	3,077 human pathways	Contains records from several source databases (Kegg, BioCyc, Reactome, NCI’s Pathway Interaction Database, Wikipathways and GO), allowing for easy integration with other NCBI databases
HumanCyc[36]	(Last updated 2017)<br>314 pathways<br>2887 reactions<br>20,830 genes<br>1,929 compounds	Subset of BioCyc for Homo Sapiens - metabolic pathways curated from publications and integrated with other databases such as gene essentiality, regulatory networks, protein features, and GO annotations. Subscription required to access most of HumanCyc and BioCyc in general beyond a limited period of free use
Pathway Commons[37]	5,772 pathways	Collects pathway and interaction data (22 different databases) and represents them in the BioPAX standard that aims to enable integration, exchange, visualization and analysis of biological pathway data

Supplementary Table 7: Open-source software packages which implement methodologies discussed in this review

Name	Type of method or algorithm	Types of data supported	Source
Connectivity Mapping[6]	Enrichment	Transcriptomics	https://clue.io/cmap
Connectivity Map[38]<br><br>gCMAP[39]	Enrichment	Transcriptomics	R packages
GOATOOLS[40]	Pathway enrichment	GO terms	Python package
GoSemSim[41]	Pathway enrichment	GO terms	R package
REVIGO[42]	GO term interpretation	GO terms	http://revigo.irb.hr/
CausalR[43]	Causal Reasoning	Transcriptomics	R package
CARNIVAL[44]	Causal Reasoning	Transcriptomics	R package
DeMAND[45]	Causal Reasoning	Transcriptomics	R package
PROTINA[46]	Causal Reasoning	Transcriptomics	R package
scikit-learn[47]	Tools for predictive data analysis	Any	Python package
MOFA[48] and MOFA+[49]	Multi omics data integration	Multi omics<br><br>MOFA+ supports single cell -omics	R and python package
TensorFlow[50]	Deep learning library	Any	Python package
PyTorch[51]	Deep learning library	Any	Python package
BMF with 'macau'[52] and 'smurff'[53]	Bayesian Matrix Factorisation	Any	Python packages
PIDGIN[54]	Target prediction tool	Chemical structure in the form of SMILES	https://github.com/BenderGroup/PIDGINv4

REFERENCES

1 A. Gaulton, L. J. Bellis, A. P. Bento, J. Chambers, M. Davies, A. Hersey, Y. Light, S. McGlinchey, D. Michalovich, B. Al-Lazikani and J. P. Overington, ChEMBL: a large-scale bioactivity database for drug discovery, *Nucleic Acids Res.*, 2012, **40**, D1100–D1107.

2 S. Kim, P. A. Thiessen, E. E. Bolton, J. Chen, G. Fu, A. Gindulyte, L. Han, J. He, S. He, B. A. Shoemaker, J. Wang, B. Yu, J. Zhang and S. H. Bryant, PubChem Substance and Compound databases, *Nucleic Acids Res.*, 2016, **44**, D1202–D1213.

3 D. S. Wishart, C. Knox, A. C. Guo, D. Cheng, S. Shrivastava, D. Tzur, B. Gautam and M. Hassanali, DrugBank: a knowledgebase for drugs, drug actions and drug targets, *Nucleic Acids Res.*, 2008, **36**, D901–D906.

4 J. Sun, N. Jeliazkova, V. Chupakhin, J.-F. Golib-Dzib, O. Engkvist, L. Carlsson, J. Wegner, H. Ceulemans, I. Georgiev, V. Jeliazkov, N. Kochev, T. J. Ashby and H. Chen, ExCAPE-DB: an integrated large scale dataset facilitating Big Data analysis in chemogenomics, *J. Cheminformatics*, , DOI:10.1186/s13321-017-0203-5.

5 T. Liu, Y. Lin, X. Wen, R. N. Jorissen and M. K. Gilson, BindingDB: a web-accessible database of experimentally determined protein-ligand binding affinities, *Nucleic Acids Res.*, 2007, **35**, D198-201.

6 J. Lamb, E. D. Crawford, D. Peck, J. W. Modell, I. C. Blat, M. J. Wrobel, J. Lerner, J.-P. Brunet, A. Subramanian, K. N. Ross, M. Reich, H. Hieronymus, G. Wei, S. A. Armstrong, S. J. Haggarty, P. A. Clemons, R. Wei, S. A. Carr, E. S. Lander and T. R. Golub, The Connectivity Map: Using Gene-Expression Signatures to Connect Small Molecules, Genes, and Disease, *Science*, 2006, **313**, 1929.

7 A. Subramanian, R. Narayan, S. M. Corsello, D. D. Peck, T. E. Natoli, X. Lu, J. Gould, J. F. Davis, A. A. Tubelli, J. K. Asiedu, D. L. Lahr, J. E. Hirschman, Z. Liu, M. Donahue, B. Julian, M. Khan, D. Wadden, I. C. Smith, D. Lam, A. Liberzon, C. Toder, M. Bagul, M. Orzechowski, O. M. Enache, F. Piccioni, S. A. Johnson, N. J. Lyons, A. H. Berger, A. F. Shamji, A. N. Brooks, A. Vrcic, C. Flynn, J. Rosains, D. Y. Takeda, R. Hu, D. Davison, J. Lamb, K. Ardlie, L. Hogstrom, P. Greenside, N. S. Gray, P. A. Clemons, S. Silver, X. Wu, W.-N. Zhao, W. Read-Button, X. Wu, S. J. Haggarty, L. V. Ronco, J. S. Boehm, S. L. Schreiber, J. G. Doench, J. A. Bittker, D. E. Root, B. Wong and T. R. Golub, A Next Generation Connectivity Map: L1000 Platform and the First 1,000,000 Profiles, *Cell*, 2017, **171**, 1437-1452.e17.

8 R. Edgar, M. Domrachev and A. E. Lash, Gene Expression Omnibus: NCBI gene expression and hybridization array data repository, *Nucleic Acids Res.*, 2002, **30**, 207–210.

9 H. Parkinson, M. Kapushesky, M. Shojatalab, N. Abeygunawardena, R. Coulson, A. Farne, E. Holloway, N. Kolesnykov, P. Lilja, M. Lukk, R. Mani, T. Rayner, A. Sharma, E. William, U. Sarkans and A. Brazma, ArrayExpress--a public database of microarray experiments and gene expression profiles, *Nucleic Acids Res.*, 2007, **35**, D747-750.

10 D. L. Svoboda, T. Saddler and S. S. Auerbach, in *Advances in Computational Toxicology: Methodologies and Applications in Regulatory Science*, ed. H. Hong, Springer International Publishing, Cham, 2019, pp. 141–157.
11 Y. Igarashi, N. Nakatsu, T. Yamashita, A. Ono, Y. Ohno, T. Urushidani and H. Yamada, Open TG-GATEs: a large-scale toxicogenomics database, *Nucleic Acids Res.*, 2015, **43**, D921-927.
12 V. Ljosa, K. L. Sokolnicki and A. E. Carpenter, Annotated high-throughput microscopy image sets for validation, *Nat. Methods*, 2012, **9**, 637.
13 M.-A. Bray, S. M. Gustafsdottir, M. H. Rohban, S. Singh, V. Ljosa, K. L. Sokolnicki, J. A. Bittker, N. E. Bodycombe, V. Dančík, T. P. Hasaka, C. S. Hon, M. M. Kemp, K. Li, D. Walpita, M. J. Wawer, T. R. Golub, S. L. Schreiber, P. A. Clemons, A. F. Shamji and A. E. Carpenter, A dataset of images and morphological profiles of 30 000 small-molecule treatments using the Cell Painting assay, *GigaScience*, , DOI:10.1093/gigascience/giw014.
14 E. Williams, J. Moore, S. W. Li, G. Rustici, A. Tarkowska, A. Chessel, S. Leo, B. Antal, R. K. Ferguson, U. Sarkans, A. Brazma, R. E. C. Salas and J. R. Swedlow, The Image Data Resource: A Bioimage Data Integration and Publication Platform, *Nat. Methods*, 2017, **14**, 775–781.
15 Recursion, RxRx: Datasets released from Recursion's automated cellular imaging and deep learning platform, to enable new machine learning tools and discover new biology., https://www.rxrx.ai/, (accessed 30 March 2021).
16 Y. Perez-Riverol, A. Csordas, J. Bai, M. Bernal-Llinares, S. Hewapathirana, D. J. Kundu, A. Inuganti, J. Griss, G. Mayer, M. Eisenacher, E. Pérez, J. Uszkoreit, J. Pfeuffer, T. Sachsenberg, Ş. Yılmaz, S. Tiwary, J. Cox, E. Audain, M. Walzer, A. F. Jarnuczak, T. Ternent, A. Brazma and J. A. Vizcaíno, The PRIDE database and related tools and resources in 2019: improving support for quantification data, *Nucleic Acids Res.*, 2019, **47**, D442–D450.
17 A. A. Saei, C. M. Beusch, A. Chernobrovkin, P. Sabatier, B. Zhang, Ü. G. Tokat, E. Stergiou, M. Gaetani, Á. Végvári and R. A. Zubarev, ProTargetMiner as a proteome signature library of anticancer molecules for functional discovery, *Nat. Commun.*, 2019, **10**, 5715.
18 K. Haug, K. Cochrane, V. C. Nainala, M. Williams, J. Chang, K. V. Jayaseelan and C. O'Donovan, MetaboLights: a resource evolving in response to the needs of its scientific community, *Nucleic Acids Res.*, 2020, **48**, D440–D444.
19 A. I. Campos and M. Zampieri, Metabolomics-Driven Exploration of the Chemical Drug Space to Predict Combination Antimicrobial Therapies, *Mol. Cell*, 2019, **74**, 1291-1303.e6.
20 T. Fuhrer, M. Zampieri, D. C. Sévin, U. Sauer and N. Zamboni, Genomewide landscape of gene-metabolome associations in Escherichia coli, *Mol. Syst. Biol.*, 2017, **13**, 907.
21 L. Litichevskiy, R. Peckner, J. G. Abelin, J. K. Asiedu, A. L. Creech, J. F. Davis, D. Davison, C. M. Dunning, J. D. Egertson, S. Egri, J. Gould, T. Ko, S. A. Johnson, D. L. Lahr, D. Lam, Z. Liu, N. J. Lyons, X. Lu, B. X. MacLean, A. E. Mungenast, A. Officer, T. E. Natoli, M. Papanastasiou, J. Patel, V. Sharma, C. Toder, A. A. Tubelli, J. Z. Young, S. A. Carr, T. R. Golub, A. Subramanian, M. J. MacCoss, L.-H. Tsai and J. D. Jaffe, A Library of Phosphoproteomic and Chromatin Signatures for Characterizing Cellular Responses to Drug Perturbations, *Cell Syst.*, 2018, **6**, 424-443.e7.
22 D. Türei, T. Korcsmáros and J. Saez-Rodriguez, OmniPath: guidelines and gateway for literature-curated signaling pathway resources, *Nat. Methods*, 2016, **13**, 966–967.
23 C. von Mering, L. J. Jensen, B. Snel, S. D. Hooper, M. Krupp, M. Foglierini, N. Jouffre, M. A. Huynen and P. Bork, STRING: known and predicted protein-protein associations, integrated and transferred across organisms, *Nucleic Acids Res.*, 2005, **33**, D433-437.
24 R. Oughtred, J. Rust, C. Chang, B. Breitkreutz, C. Stark, A. Willems, L. Boucher, G. Leung, N. Kolas, F. Zhang, S. Dolma, J. Coulombe-Huntington, A. Chatr-aryamontri, K. Dolinski and M. Tyers, The BioGRID database: A comprehensive biomedical resource of curated protein, genetic, and chemical interactions, *Protein Sci. Publ. Protein Soc.*, 2021, **30**, 187–200.
25 E. L. Huttlin, L. Ting, R. J. Bruckner, F. Gebreab, M. P. Gygi, J. Szpyt, S. Tam, G. Zarraga, G. Colby, K. Baltier, R. Dong, V. Guarani, L. P. Vaites, A. Ordureau, R. Rad, B. K. Erickson, M. Wühr, J. Chick, B. Zhai, D. Kolippakkam, J. Mintseris, R. A. Obar, T. Harris, S. Artavanis-Tsakonas, M. E. Sowa, P. DeCamilli, J. A. Paulo, J. W. Harper and S. P. Gygi, The BioPlex Network: A Systematic Exploration of the Human Interactome, *Cell*, 2015, **162**, 425–440.
26 A. Kamburov, C. Wierling, H. Lehrach and R. Herwig, ConsensusPathDB--a database for integrating human functional interaction networks, *Nucleic Acids Res.*, 2009, **37**, D623-628.
27 A. K. Wong, A. Krishnan and O. G. Troyanskaya, GIANT 2.0: genome-scale integrated analysis of gene networks in tissues, *Nucleic Acids Res.*, 2018, **46**, W65–W70.
28 R. Goel, H. C. Harsha, A. Pandey and T. S. K. Prasad, Human Protein Reference Database and Human Proteinpedia as resources for phosphoproteome analysis, *Mol. Biosyst.*, 2012, **8**, 453–463.
29 H. Hermjakob, L. Montecchi-Palazzi, C. Lewington, S. Mudali, S. Kerrien, S. Orchard, M. Vingron, B. Roechert, P. Roepstorff, A. Valencia, H. Margalit, J. Armstrong, A. Bairoch, G. Cesareni, D. Sherman and R. Apweiler, IntAct: an open source molecular interaction database, *Nucleic Acids Res.*, 2004, **32**, D452–D455.
30 M. Zitnik, S. Maheshwari and J. Leskovec, Stanford Biomedical Network Dataset Collection, http://snap.stanford.edu/biodata/, (accessed 30 March 2021).
31 A. Fabregat, S. Jupe, L. Matthews, K. Sidiropoulos, M. Gillespie, P. Garapati, R. Haw, B. Jassal, F. Korninger, B. May, M. Milacic, C. D. Roca, K. Rothfels, C. Sevilla, V. Shamovsky, S. Shorser, T. Varusai, G. Viteri, J. Weiser, G. Wu, L. Stein, H. Hermjakob and P. D'Eustachio, The Reactome Pathway Knowledgebase, *Nucleic Acids Res.*, 2018, **46**, D649–D655.
32 M. Kanehisa and S. Goto, KEGG: Kyoto Encyclopedia of Genes and Genomes, *Nucleic Acids Res.*, 2000, **28**, 27–30.
33 D. N. Slenter, M. Kutmon, K. Hanspers, A. Riutta, J. Windsor, N. Nunes, J. Mélius, E. Cirillo, S. L. Coort, D. Digles, F. Ehrhart, P. Giesbertz, M. Kalafati, M. Martens, R. Miller, K. Nishida, L. Rieswijk, A. Waagmeester, L. M. T. Eijssen, C. T.

Evelo, A. R. Pico and E. L. Willighagen, WikiPathways: a multifaceted pathway database bridging metabolomics to other omics research, *Nucleic Acids Res.*, 2018, **46**, D661–D667.
34 M. Ashburner, C. A. Ball, J. A. Blake, D. Botstein, H. Butler, J. M. Cherry, A. P. Davis, K. Dolinski, S. S. Dwight, J. T. Eppig, M. A. Harris, D. P. Hill, L. Issel-Tarver, A. Kasarskis, S. Lewis, J. C. Matese, J. E. Richardson, M. Ringwald, G. M. Rubin and G. Sherlock, Gene Ontology: tool for the unification of biology, *Nat. Genet.*, 2000, **25**, 25–29.
35 L. Y. Geer, A. Marchler-Bauer, R. C. Geer, L. Han, J. He, S. He, C. Liu, W. Shi and S. H. Bryant, The NCBI BioSystems database, *Nucleic Acids Res.*, 2010, **38**, D492–D496.
36 M. Trupp, T. Altman, C. A. Fulcher, R. Caspi, M. Krummenacker, S. Paley and P. D. Karp, Beyond the genome (BTG) is a (PGDB) pathway genome database: HumanCyc, *Genome Biol.*, 2010, **11**, O12.
37 E. G. Cerami, B. E. Gross, E. Demir, I. Rodchenkov, Ö. Babur, N. Anwar, N. Schultz, G. D. Bader and C. Sander, Pathway Commons, a web resource for biological pathway data, *Nucleic Acids Res.*, 2011, **39**, D685–D690.
38 P. Shannon, *ConnectivityMap*, 2020.
39 T. Sandmann, S. K. Kummerfeld, R. Gentleman and R. Bourgon, gCMAP: user-friendly connectivity mapping with R, *Bioinformatics*, 2014, **30**, 127–128.
40 D. V. Klopfenstein, L. Zhang, B. S. Pedersen, F. Ramírez, A. Warwick Vesztrocy, A. Naldi, C. J. Mungall, J. M. Yunes, O. Botvinnik, M. Weigel, W. Dampier, C. Dessimoz, P. Flick and H. Tang, GOATOOLS: A Python library for Gene Ontology analyses, *Sci. Rep.*, 2018, **8**, 10872.
41 G. Yu, F. Li, Y. Qin, X. Bo, Y. Wu and S. Wang, GOSemSim: an R package for measuring semantic similarity among GO terms and gene products, *Bioinforma. Oxf. Engl.*, 2010, **26**, 976–978.
42 F. Supek, M. Bošnjak, N. Škunca and T. Šmuc, REVIGO Summarizes and Visualizes Long Lists of Gene Ontology Terms, *PLOS ONE*, 2011, **6**, e21800.
43 G. Bradley and S. J. Barrett, CausalR: extracting mechanistic sense from genome scale data, *Bioinformatics*, 2017, **33**, 3670–3672.
44 A. Liu, P. Trairatphisan, E. Gjerga, A. Didangelos, J. Barratt and J. Saez-Rodriguez, From expression footprints to causal pathways: contextualizing large signaling networks with CARNIVAL, *Npj Syst. Biol. Appl.*, 2019, **5**, 1–10.
45 J. H. Woo, Y. Shimoni, W. S. Yang, P. Subramaniam, A. Iyer, P. Nicoletti, M. Rodríguez Martínez, G. López, M. Mattioli, R. Realubit, C. Karan, B. R. Stockwell, M. Bansal and A. Califano, Elucidating Compound Mechanism of Action by Network Perturbation Analysis, *Cell*, 2015, **162**, 441–451.
46 H. Noh, J. E. Shoemaker and R. Gunawan, Network perturbation analysis of gene transcriptional profiles reveals protein targets and mechanism of action of drugs and influenza A viral infection, *Nucleic Acids Res.*, 2018, **46**, e34.
47 F. Pedregosa, G. Varoquaux, A. Gramfort, V. Michel, B. Thirion, O. Grisel, M. Blondel, P. Prettenhofer, R. Weiss, V. Dubourg, J. Vanderplas, A. Passos and D. Cournapeau, Scikit-learn: Machine Learning in Python, *Mach. Learn. PYTHON*, 6.
48 R. Argelaguet, B. Velten, D. Arnol, S. Dietrich, T. Zenz, J. C. Marioni, F. Buettner, W. Huber and O. Stegle, Multi-Omics Factor Analysis—a framework for unsupervised integration of multi-omics data sets, *Mol. Syst. Biol.*, 2018, **14**, e8124.
49 R. Argelaguet, D. Arnol, D. Bredikhin, Y. Deloro, B. Velten, J. C. Marioni and O. Stegle, MOFA+: a statistical framework for comprehensive integration of multi-modal single-cell data, *Genome Biol.*, 2020, **21**, 111.
50 M. Abadi, P. Barham, J. Chen, Z. Chen, A. Davis, J. Dean, M. Devin, S. Ghemawat, G. Irving, M. Isard, M. Kudlur, J. Levenberg, R. Monga, S. Moore, D. G. Murray, B. Steiner, P. Tucker, V. Vasudevan, P. Warden, M. Wicke, Y. Yu and X. Zheng, in *12th USENIX Symposium on Operating Systems Design and Implementation (OSDI 16)*, 2016, pp. 265–283.
51 A. Paszke, S. Gross, F. Massa, A. Lerer, J. Bradbury, G. Chanan, T. Killeen, Z. Lin, N. Gimelshein, L. Antiga, A. Desmaison, A. Köpf, E. Yang, Z. DeVito, M. Raison, A. Tejani, S. Chilamkurthy, B. Steiner, L. Fang, J. Bai and S. Chintala, PyTorch: An Imperative Style, High-Performance Deep Learning Library, *ArXiv191201703 Cs Stat*.
52 J. Simm, A. Arany, P. Zakeri, T. Haber, J. K. Wegner, V. Chupakhin, H. Ceulemans and Y. Moreau, Macau: Scalable Bayesian Multi-relational Factorization with Side Information using MCMC, *ArXiv150904610 Stat*.
53 T. V. Aa, I. Chakroun, T. J. Ashby, J. Simm, A. Arany, Y. Moreau, T. L. Van, J. F. G. Dzib, J. Wegner, V. Chupakhin, H. Ceulemans, R. Wuyts and W. Verachtert, SMURFF: a High-Performance Framework for Matrix Factorization, *ArXiv190402514 Cs Stat*.
54 L. H. Mervin, A. M. Afzal, G. Drakakis, R. Lewis, O. Engkvist and A. Bender, Target prediction utilising negative bioactivity data covering large chemical space, *J. Cheminformatics*, 2015, **7**, 51.
